# New Carbonic Anhydrase-II Inhibitors from Marine Macro Brown Alga *Dictyopteris hoytii* Supported by In Silico Studies

**DOI:** 10.3390/molecules26237074

**Published:** 2021-11-23

**Authors:** Kashif Rafiq, Ajmal Khan, Najeeb Ur Rehman, Sobia Ahsan Halim, Majid Khan, Liaqat Ali, Abdullah Hilal Al-Balushi, Haitham Khamis Al-Busaidi, Ahmed Al-Harrasi

**Affiliations:** 1 Natural & Medical Sciences Research Center, University of Nizwa, P.O. Box 33, Nizwa 616, Oman; kashifrafiq609@gmail.com (K.R.); ajmalkhan@unizwa.edu.om (A.K.); sobia_halim@unizwa.edu.om (S.A.H.); majidk166@yahoo.com (M.K.); malikhejric@gmail.com (L.A.); 2 H. E. J. Research Institute of Chemistry, International Center for Chemical and Biological Sciences, University of Karachi, Karachi 75270, Pakistan; 3 Department of Chemistry, University of Mianwali, Mianwali 42200, Pakistan; 4 Oman Animal and Plant Genetic Resources Center, P.O. Box 92, Muscat 123, Oman; abdullah.albalushi@oapgrc.gov.om (A.H.A.-B.); Haihtam.AlBusaidi@oapgrc.gov.om (H.K.A.-B.)

**Keywords:** *Dictyopteris hoytii*, carbonic anhydrase-II inhibitors, NMR spectroscopy, molecular docking, ADMET prediction

## Abstract

In continuation of phytochemical investigations of the methanolic extract of *Dictyopteris hoytii,* we have obtained twelve compounds (**1**–**12**) through column chromatography. Herein, three compounds, namely, dimethyl 2-bromoterepthalate (**3**), dimethyl 2,6-dibromoterepthalate (**4**), and (*E*)-3-(4-(dimethoxymethyl)phenyl) acrylic acid (**5**) are isolated for the first time as a natural product, while the rest of the compounds (**1**, **2**, **6**–**12**) are known and isolated for the first time from this source. The structures of the isolated compounds were elucidated by advanced spectroscopic 1D and 2D NMR techniques including ^1^H, ^13^C, DEPT, HSQC, HMBC, COSY, NEOSY, and HR-MS and comparison with the reported literature. Furthermore, eight compounds (**13**–**20**) previously isolated by our group from the same source along with the currently isolated compounds (**1**–**12**) were screened against the CA-II enzyme. All compounds, except **6**, **8**, **14**, and **17**, were evaluated for *in vitro* bovine carbonic anhydrase-II (CA-II) inhibitory activity. Eventually, eleven compounds (**1**, **4**, **5**, **7**, **9**, **10**, **12**, **13**, **15**, **18**, and **19**) exhibited significant inhibitory activity against CA-II with IC_50_ values ranging from 13.4 to 71.6 μM. Additionally, the active molecules were subjected to molecular docking studies to predict the binding behavior of those compounds. It was observed that the compounds exhibit the inhibitory potential by specifically interacting with the ZN ion present in the active site of CA-II. In addition to ZN ion, two residues (His94 and Thr199) play an important role in binding with the compounds that possess a carboxylate group in their structure.

## 1. Introduction

In recent years, more than 200,000 eukaryotic marine species have been validated, among which, algae contributed nearly 44,000 well described species [1]. Among algae, macroalgae (seaweeds) serve as a novel source of compounds, due to their use in traditional medicine and nutritional importance [2]. The chemical composition varies considerably due to both environmental condition and genetic differences among species [3]. These are valuable due to their high content in compounds with different biological activities, including both complex organic compounds and primary and secondary metabolites [3,4]. Moreover, these seaweeds can be used as a source of biofuels due to their high growth rate, high photosynthetic efficiency, no competition with food crops, high carbohydrate content, and no requirement of land area for cultivation [5]. Within the marine environment, seaweeds are a rich reservoir of structurally diverse natural products (NPs), mostly belonging to the class of phenolic compounds, terpenoids, tannins, peptides, polysaccharides, vitamins, iodine, and brominated organic products, with potential applications for drug discovery [6,7,8]. The genus *Dictyopteris* (brown algae) is among the few odoriferous types of seaweed, which are structurally similar to sexual attractants and act as odoriferous compounds due to their C11-hydrocarbons [9].

*Dictyopteris hoytii* W.R. Taylor belongs to genus *Dictyopteris* (family: Dictyotaceae), comprised of 35 species, and known by its characteristic “ocean smell” [10]. The genus *Dictyopteris* is an important group of marine seaweeds and is widely distributed in tropical, sub-tropical, and temperate regions [11]. Since the middle of the 20th century, the genus has gained attention and proved to be a great source of structurally diverse secondary metabolites including C-24 epimers of Δ^28^-24-hydroxy stigmastane-type steroids [12], brominated selinane sesquiterpenes [13], oplopane-type sesquiterpenes [14], brominated terephthale [15], germacrane-type sesquiterpenes [16], meroterpenoids [17], cadinane sesquiterpenes [18], sesquiterpene-substituted benzoquinone derivatives [19], norsesquiterpenes [20], C11 hydrocarbons [21], and C11 sulfur-containing compounds [22] of either normal or mixed biogenesis. Various secondary metabolites have been obtained from *Dictyopteris* species with several potential biological activities including neuroprotective [23], antibacterial, antioxidant [24], cytotoxic [25], α-glucosidase inhibitory [15], anti-inflammatory [22], anti-cancer [12], algicidal [21], piscicidal [22], anti-feedant [14], antimicrobial, and anti-herbivory properties [11,13].

The carbonic anhydrases (CAs, EC 4.2.1.1, metal ion-dependent enzymes), are well-known for their ability to catalyze the conversion of CO_2_ to bicarbonate, and protons [26]. CAs are found in a lot of living organisms and play an important role in various physiological processes related to respiration, and transport of CO_2_/bicarbonate between metabolizing tissues, and the lungs, maintenance of pH, and CO_2_ homeostasis, tumorigenicity, electrolyte excretion in different tissues/organs, bone resorption, biosynthetic reactions, calcification, and several physiological or pathological processes [26,27,28]. In algae, plants and some bacteria, CA play a crucial role in photosynthesis and other biosynthetic reactions [29]. Out of 16 reported CA isoforms, only 12 are catalytically active and vary with respect to their location, kinetic properties, and inhibitor profiles. The over expression of CA-II is associated with epilepsy, glaucoma, cancer, obesity, leukemia, and cystic fibrosis [27,29]. Recently, essential oils (EOs) have been reported as possible source of CA inhibitors (CAIs), particularly against the mitochondrial isoform VA, which is a potential therapeutic target to treat obesity [30].

**Figure 1:**
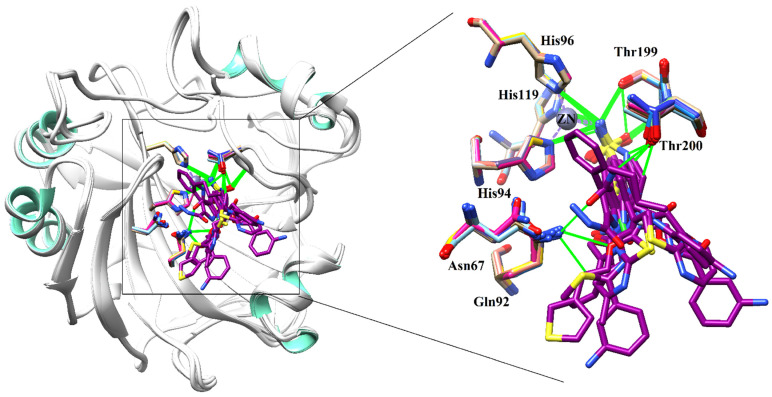
The superimposed conformations of several 3D-Structure of Human CA-II are shown in complex with their cognate ligands (PDB codes: 2FOU, 3B4F, 3HS4, 3K34, 4FPT, 4IZW, 4KNI, 4KNJ, 5BYI, 6G6T, 6S6G, and 7JNR). The ligands are presented in magenta stick model, and H-bonds are depicted in green lines.

Several sulfonamide derivatives (e.g., acetazolamide, dichlorphenamide, methazolamide, ethoxzolamide, etc.) were designed as CA-II inhibitors, which were used systemically in antiglaucoma therapy [26]. The active site of CA-II contains a zinc ion with catalytically active residues (Thr199, Glu106, His 64, and His 94). To gain more insights of chemical features of inhibitors which are essential in binding with the protein, we selected some CA-II-ligand complexes. By structural alignment of those selected protein–ligand complexes, we extracted common interaction features which suggested that Asn67, Gln92, His94, His96, His119, Thr199, and Thr200 are critical residues in stabilizing the ligand within the active site of CA-II (Figure 1).

Considering our interest in the identification of bioactive metabolites from marine macro-algae, we isolated twelve compounds (**1**–**12**) from the methanolic extract of *D. hoytii* for the first time, whereas compounds **3**–**5** were isolated for the first time as NPs. However, this research also supports novel findings of volatile constituents of essential oil (extracted from methanol fraction) through column chromatography.

## 2. Results and Discussion

### 2.1. Structure Elucidation of Compounds ***1***–***12***

**Figure 2:**
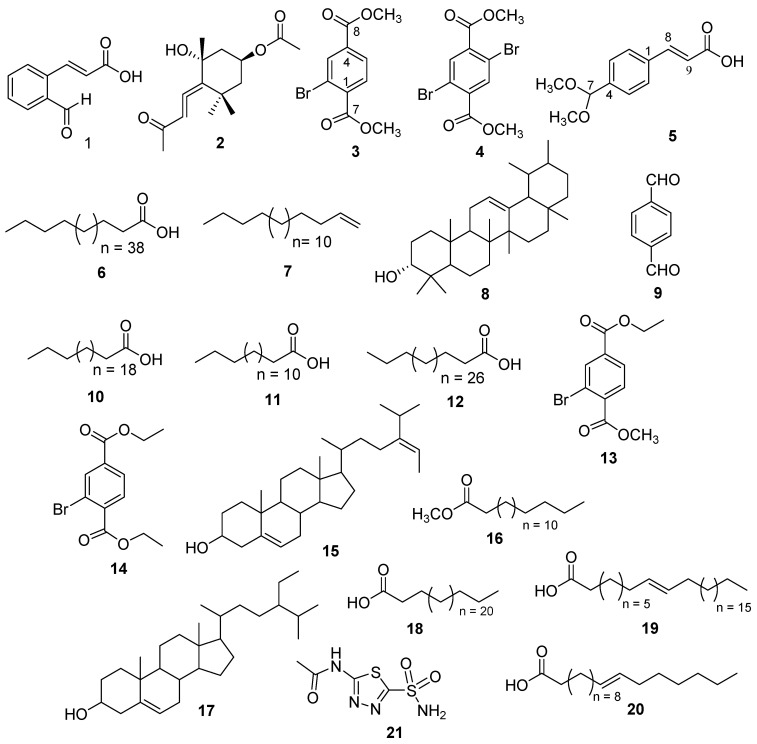
Structures of the compounds **1**–**20** isolated from *D. hoytii* and acetazolamide (**21**).

The molecular formula (C_10_H_9_BrO_4_) of compound **3** was determined from high resolution-electrospray ionization (HR-ESI)-MS spectrum having a pseudomolecular ion at *m*/*z* 294.9581 [^79^BrM + Na]^+^ (Calcd. 294.9582), and 296.9560 [^81^BrM+Na]^+^ (Cald. 296.9562). The ^1^H- and ^13^C-NMR spectra of **3** were identical to those of a 2-bromoethylmethylbenzene-1,4-dioate (**13**), previously isolated from the same species [15] with the exception of a methoxy in **3** instead of ethoxy group in **13**. Compound **3** was isolated as colorless powder (Figure 2) from the methanolic extract of *D. hoytii*. The IR spectrum exhibited the presence of carbonyl ester (1735, 1654 cm^−1^), and aromatic ring (1598, 1510, and 1430 cm^−1^) in the molecule. The data obtained from ^1^H NMR spectrum of **1** displayed signals assigned to three aromatic protons at δ_H_ 8.29 (1H, d = 1.8 Hz, H-3), 7.98 (1H, dd = 7.8, 1.8 Hz, H-5), and 7.79 (1H, d = 7.8 Hz, H-6), which was further confirmed by ^13^C NMR spectra at δ 135.1 (C-3), 128.0 (C-5), and 131.0 (C-6). The proton at H-5 was found ortho to H-6 due to their large coupling constants (J > 7 Hz), while the low coupling constant of 1.8 Hz between H-3 and H-5 revealed that H-3 was meta to H-5, which was further confirmed by the ^1^H-^1^H COSY correlations between H-5 and H-6 (Figure 2). In addition, ^1^H-NMR spectrum also displayed one singlet at 3.93 (6H, s, OCH_3_) corresponding to the presence of two methoxy groups and their HMBC correlation with δ_C_ 166.1 (C-7), and 164.9 (C-8) further confirmed the presence of ester groups. The ^13^C-NMR spectrum disclosed 10 signals which were classified with the aid of distortionless enhancement by polarization transfer (DEPT) and heteronuclear single-quantum correlation (HSQC) experiments as a presence of two methoxy, three aromatic methines, two carbonyls, and three quaternary carbons. The downfield signals observed in the spectra at δ_C_ 166.1, 164.9, 52.7, and 52.6 support the presence of these functional groups in the molecule. Furthermore, the correlations observed in HMBC of H-3 with C-2, C-4, and C-8; H-5 with C-4, C-6, and C-8; H-6 with C-5, C-4, C-7, and C-1 further supported the presence of methyl esters at the para positions. The above data were compatible with a benzyl skeleton substituted with two methyl esters at para positions and one ortho substituted bromine, which was further supported by ^1^H–^1^H correlation spectroscopy (COSY) and heteronuclear multiple bond correlation (HMBC) experiment. Based on these facts, compound **3** was assigned as dimethyl 2-bromoterepthalate.

Compound **4** was isolated as a white powder with a molecular formula, C_10_H_8_Br_2_O_4_, established by positive HR-ESI-MS at *m*/*z* 367.8938 ([M + H_2_O]^+^ (Calcd. 367.8895) (Figure 2). The ^1^H- and ^13^C-NMR spectra of **4** closely resembled those of compound **3** with the only difference being additional bromine at C-4 attached to the benzyl ring. Due to symmetry in the molecule, one downfield signal at δ 8.04 with double integration (2H, s, H-3/6) was observed in ^1^H-NMR spectra assigned to two aromatic protons H-3/6 and this was further confirmed by ^13^C-NMR spectra at δ 166.3 (C-7/8). Moreover, the ^1^H-NMR spectrum displayed two signals at 3.96 (3H, s), and 3.88 (3H, s), which corresponded to the presence of two methoxy groups and their HMBC correlations with C-7/8 further confirmed the presence of dimethyl esters (Figure 2). The HMBC correlations of H-3 to C-2, C-4, C-1, and C-8 and H-5 to C-6, C-4, C-8, and C-1 further indicated the presence and position of ester groups at C-1 and C-4 in the molecule. Because of these advance spectroscopic techniques and above observations, the structure of compound **4** was designated as dimethyl 2,6-dibromoterepthalate.

**Figure 3:**
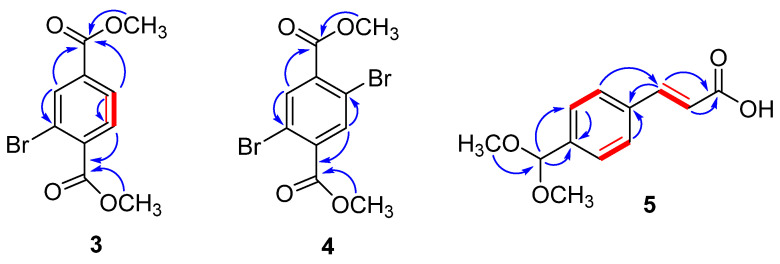
Key H→C HMBC and H—H COSY correlations of compounds **3**, **4**, and **5**.

Compound **5** was isolated as off-white needles. The positive HR-ESI-MS spectrum exhibited a pseudomolecular ion at *m*/*z* 230.9619 [M + Na]^+^ (Calcd. 231.0638) indicating a molecular formula of C_12_H_14_O_4_. IR spectrum showed absorption peaks at 567 (olefin), 1590, 1514, 1420 (aromatic), and 3200–3500 cm^−1^ (COOH). The ^1^H-NMR spectrum exhibited signals assigned to two aromatic protons with double integration at δ 7.40 (2H, d, *J* = 8.4 Hz, H-2/6) and 7.67 (2H, d, *J* = 8.4 Hz, H-3/5) and this was further confirmed by ^13^C-NMR spectra (δ 127.9 (C-3/5) and 127.0 (C-2/6). The ^1^H-NMR spectrum (Figure 2) exhibited resonances at δ 3.23 (6H, s, 2 × OCH_3_), and 5.38 (1H, s, H-7) showing the presence of two methoxy groups and one anomeric carbon. The large coupling constant (J = 15.0 Hz) observed for H-8 (δ_H_ 7.56) and H-9 (δ_H_ 6.52) and the chemical shifts of C-8 (δ_C_ 143.0) and C-9 (δ_C_ 129.9) were indicative of a 8E,9E configuration of the double bonds [31]. The downfield signal at δ 167.3 in ^13^C-NMR spectrum and the interaction of olefinic proton with carbonyl in HMBC correlation confirmed the presence of acrylic acid in the molecule. The large coupling constant values of aromatic protons between H-2/3 and H-5/6 further clarified the para-substitution of acrylic acid. ^13^C-NMR spectrum (BB, and DEPT) showed resonances for eleven carbons including two methoxy, one double bond, four aromatic, one anomeric, and three quaternary carbons. Structure of the compound was further confirmed by using 2D-NMR technique (HSQC, HMBC, and COSY). Key HMBC and COSY interactions in compound **5** are shown in Figure 3. Compound **5** ((E)-3-(4-(dimethoxymethyl)phenyl)acrylic acid) was reported as synthetic derivative, however, isolated from the natural source for the first time. Compounds **3**–**5**, to the best of author’s knowledge, have not been described earlier as natural products, while synthesized previously by many researchers [32,33,34,35,36,37].

The structures of the known compounds including (E)-3-(2-formylphenyl)acrylic acid (**1**) [38,39], apo-9-fucoxanthinone (**2**) [40,41], pentatetracontanoic acid (**6**) [42,43], octadec-1-ene (**7**) [44], epi-amyrine (**8**) [45,46], terephthalaldehyde (**9**) [47,48], tricosylic acid (**10**) [49,50], hexadecanoic acid (**11**), and lacceroic acid (**12**) [51,52] were determined upon comparing their spectral data with those reported in the literature (Figure 2). Other compounds like ethyl methyl 2-bromobenzene 1,4-dioate (**13**), diethyl 2-bromobenzene 1,4-dioate (**14**), fucosterol (**15**), n-hexadecanoic acid, methyl ester (**16**), β-sitosterol (**17**), cerotic acid (**18**), n-octacos-9-enoic acid (**19**), and 11-eicosenoic acid (**20**) were previously reported from the same species with α-glucosidase inhibitory activity [15]. Compound **1** was isolated for the first time from the marine species; however, it had been previously isolated from the AcOEt layer of ozonolysis of mononuclear phenols [38].

### 2.2. Carbonic Anhydrase-II Inhibition and Structural-Activity Relationship

In the fields of medicine, drug discovery is under increased pressure to recognize more appropriate secondary metabolites as starting points for drug development and searching for new compounds as chemical starting points for optimization is one of the key challenges in drug discovery research. To search novel inhibitors, all the isolated compounds (except compounds **6**, **8**, **14**, and **17**) were evaluated for CA-II inhibitory potential. Subsequently, most of the compounds demonstrated moderate to significant inhibition with IC_50_ values in range from 13.4 to 71.6 μM, as compared to standard inhibitor acetazolamide (IC_50_ = 18.2 μM, Table 1). In the preliminary screening, eleven compounds (**1**, **4**, **5**, **7**, **9**, **10**, **12**, **13**, **15**, **18**, and **19**) were found active, while compounds **2**, **3**, **11**, **16**, and **20** displayed less than 50% inhibition, however, due to paucity of compounds **6**, **8**, **14**, and **17**, they were not evaluated. The compounds **4**, **5**, **7**, **12**, and **18** exhibited excellent inhibitory activity, whereas compounds **13**, **15**, and **19** displayed comparable inhibition as compared to standard, while compounds **1**, **9**, and **10** showed moderate inhibitory activities (Table 1).

When we compared the activity of most active compounds with clinically standard inhibitor acetazolamide (IC_50_ =18.2 ± 1.23 μM), the compounds **4**, **5**, **7**, **12**, and **18** showed better potency against carbonic anhydrase II enzyme. Comparing compound **1** with **5**, higher inhibition of **5** (17.7 ± 2.01 μM) than **1** (44.9 ± 1.10 μM) may be due to the presence of dimethoxymethyl at *para* position to the acrylic acid. Our results match with the reported data of Innocenti et al. [53], who studied some phenolic compounds including p-coumaric acid, caffeic acid, and ferulic acid, similar in structure to compounds **1** and **5**, against CA-II and showed excellent activity in the range of 0.98–2.4 μM. Higher inhibition of these compounds than **1** and **5** may be due to the replacement of –OH with –OCH_3_ groups and their intermolecular arrangements. Comparing compounds **3** (inactive), **4** (13.4 μM), **13** (39.5 μM), and **14** (inactive), compound **4** displayed higher inhibition which may be possibly due to the presence of two bromines at *ortho* positions to the acetate groups of benzene ring. Among straight chain acids, compound **12** (11.6 μM) determined promising inhibition as compared to **10** (71.6 μM), **18** (17.0 μM), and **19** (24.3 μM), which could be attributed due to the presence of large number of carbons. A slight variation in activities between **18** and **19** was observed, which can be assigned to the absence of double bond in **18**. The present data offer new insights on possible novel classes of CA II inhibitors based on natural products, possessing a range of chemical structures do not present in the clinical inhibitors with pharmacological applications, such as the sulfonamides, acetazolamide, and sulfamates. In addition, the CA-II inhibitory activity of all the active compounds are reported for the first time.

### 2.3. Molecular Docking of Carbonic Anhydrase-II Inhibitors

Eleven compounds (**1**, **5**, **7**, **9**–**10**, **12**–**13**, **15**, **18**, and **19**) exhibited significant inhibitory potential against CA-II in an in vitro experiment. Therefore, the binding mechanism of the active hits was predicted in the active site of CA-II through a molecular docking strategy. When docked at the active site of CA-II, the most active compound, **12**, demonstrated excellent binding with ZN ion in the center of the active site. The carboxylate moiety of lacceroic acid (**12**) formed strong ionic interactions with ZN ion and accepted a hydrogen bond from the side chains of His94, Thr199, and Thr200. Additionally, the main chain amino nitrogen of Thr199 also mediated the H-bond with the carboxylate group of the compound. This suggests that the carboxylate moiety is responsible for the binding, in turn, for inhibitory activity of the compound. The docked conformation of **12** depicted that the long alkyl chain of the compound covered the entrance of the active site completely; however, the solvent was found partially exposed. After compound **12**, compound **4** demonstrated good inhibitory activity. The binding mode of compound **4** revealed that one of the carbonyl moieties of the compound binds with ZN ion through ionic interaction and with His94 via the H-bond; however, the other carbonyl group did not show any interaction. Due to the lack of a polar group or warhead in the structure of compound **7**, this molecule only interacted with the side chain of His94 through hydrophobic interaction; however, the compound was also found to be accommodated nicely in the active site. The binding mode of compound **18** (cerotic acid) resembled the docked orientation of compound **12**. The carboxyl group of **18** also formed ionic interaction with ZN ion, and H-bonds with the side chain and main chain nitrogen of His94 and Thr199, respectively, while the alkyl chain of this compound remained solvent exposed. Similarly, the carboxylate group of **5** was involved in the bidentate interaction with ZN ion and H-bonding with the side chains of His94, His96, and Thr199. 

The docked views of compounds **19**, **15**, and **13** revealed that the carboxylate group of **19** bounds with ZN ion by ionic interactions, and additionally formed H-bonds with the side chain of Thr199; however, like compound **18**, the alkyl chain of **19** also remained solvent exposed. The mode of binding of **15** (fucosterol) showed that the –OH group of **15** mediated the H-bond with the side chain of His94, and ionic interaction with ZN ion, whereas the rest of the structure of **15** remained surface exposed. The docked conformation of compound **13** was found quite similar to that of the docked orientation of compound **4**. Like compound **4**, one of the carbonyl groups of **13** formed ionic interaction with ZN ion, and an H-bond with the side chain of His94. Similarly, Thr199 provided the H-bond to the formyl and the carboxylate groups of **1**, one of the aldehyde groups of compound **9**, and the carboxylate group of compound **10**, while these compounds interacted with ZN ion through ionic interaction. Moreover, the least active molecule, **10**, also demonstrated an H-bond with the side chain of His94. However, the compound **10** was found mostly solvent exposed. The binding mode of all these compounds revealed that His94 and Thr199 play an important roles in the stabilization of these compounds in the active site of CA-II. However, several residues (His4, Trp5, Leu60, Asn62, His64, Asn67, and Phe131) at the entrance of the active site, give support to the surface-exposed atoms of these ligands.

**Figure 4:**
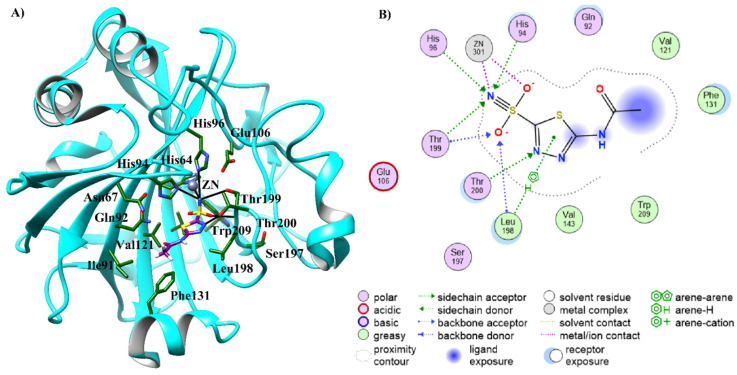
(**A**) The docked view of acetazolamide (positive control, presented in purple stick model) in the active site of CA-II after docking. The protein is shown in cyan ribbon model, the active site residues are presented in the green stick model. H-bonds are depicted in black lines. (**B**) The 2D view of binding interactions of acetazolamide is shown in the active site of CA-II.

**Figure 5:**
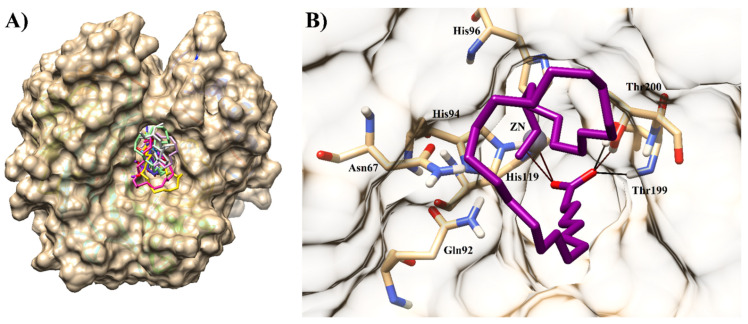
(**A**) The binding mode of all the docked compounds are shown. The protein is shown in surface presentation and the ligands are depicted in stick model. (**B**) The docked view of the most active inhibitor (**12**) is shown in the active site of CA-II. The protein is shown in surface model; interacting residues are presented in tan stick model; H-bonds are depicted in black lines.

The positive control, acetazolamide, a high affinity inhibitor of CA-II was also docked at the active site of CA-II among other inhibitors. The docked view of the compound depicted that the sulfonamide group of acetazolamide interacted with ZN ion through metallic and ionic interactions, and with His94, His96, and Thr199 via H-bonds, while the carbonyl moiety of the control interacted with a surrounding solvent molecule (Figure 4). The docking score of acetazolamide (docking score = −9.47 kcal/mol) was found to be lower than the docking scores of compounds **12** (−7.40 kcal/mol) and **19** (−9.66 kcal/mol), while higher than the docking scores of rests of the compounds. The docking scores and binding interactions of the docked compounds are shown in Table 2. We observed that the docking scores of the structurally similar compounds with long alkyl chains (**12**, **7**, **18**, **19**, and **20**) were higher (in range of >−9 kcal/mol) than that of the docking scores of small molecules with ring structures (**1**, **4**, **5**, **9**, and **13**, docking score in range of >−5kcal/mol) inhibitors, showing a good trend of correlation with the biological activities of the compounds. The binding modes of all the compounds are presented in Figure 5.

### 2.4. Pharmacokinetic Prediction of Compounds ***1***, ***3***, ***5***, ***7***, ***9***–***10***, ***12***–***13***, ***15***, ***18***, and ***19***

The pharmacokinetic properties of compounds **1**, **3**, **5**, **7**, **9–10**, **12–13**, **15**, **18**, and **19** were predicted by the SwissADME server and compared with the pharmacokinetic behavior of reference ligand and acetazolamide. All the compounds possessed good physicochemical properties. Compounds **1**, **4–5**, **9**, and **13** possessed high gastrointestinal absorption. Which none of the compounds acted as a substrate for P-glycoprotein. Like reference ligand and positive control, compounds **7**, **10**, **12**, **15**, **18**, and **19** did not possess blood–brain–barrier permeability. The compounds demonstrated a low to high partition coefficient (Log P_o/w_). Most of the compounds were not skin permeable. None of the compounds inhibited cytochrome P450 (CYP) 2C9, CYP2D6, and CYP3A4, whereas compounds **4**, **7**, **9–10**, and **13** depicted inhibitory potential for CYP1A2, and compounds **4** and **13** showed inhibition of CYP2C19. All the compounds followed the Lipinski rule of five, thus demonstrated drug-likeness properties. None of the compound showed PAINs alert. Only compounds **4** and **13** and the reference ligand possessed lead-likeness properties. The synthetic accessibility (SA) of the compounds was in range of 1.00 to >6. The predicted ADMET properties of the compounds are tabulated in Table S1.

## 3. Material and Methods

This study comprises of extraction and isolation of bioactive compounds from *D. hyotii* and determination of CA-II inhibitory activity. The molecular docking studies and prediction of pharmacokinetic properties were performed computationally. The computational results were validated by in vitro testing of active compounds against most suitable predicted target.

### 3.1. General Instrumentation

High-resolution electrospray ionization mass spectrometry (HR-ESI-MS) was documented on Agilent Technologies (6530 Accurate Mass, Q-TOF LC/MS). ^1^H NMR (Nuclear Magnetic Resonance) spectra were recorded on 600 MHz and ^13^C NMR on 150 MHz spectrometers using the solvent peak as internal reference (CDCl_3_, δ_H_: 7.26; δ_C_: 77.0) (CD_3_OD, δ_H_: 4.87; δ_C_: 48.5). NMR data was reported as chemical shift (δ) in ppm; multiplicities are as singlet (s), doublet (d), triplet (t), doublet of doublet (dd), multiplet (m); coupling constants (J) are in hertz (Hz). Optical rotation ([α]^25^_D_) was measured on a KRUSS P3000 polarimeter (A. Kruss Optronic, Germany). Column chromatography (CC) was carried out by using silica gel of the selected particle size of 100–200 mesh. Visualizations of the thin layer chromatographic plates were attained under the UV light at 254 and 366 nm, and by spraying with the ceric sulphate reagent. Solvents for column chromatography (ethyl acetate, EtOAc), methanol, dichloromethane, and *n*-hexane) were of technical grade and distilled prior to use. Microplate reader (Bio-Rad, Molecular Devices, CA, USA) was used for carbonic anhydrase activity.

### 3.2. Sample Collection and Identification

The seaweed fresh samples of *D. hoytii* were collected (April, 2017) from coastal region of Raysut (16°59′10.04″ N, 54°1′58.59″ E), Dhofar, Oman, and were provided by Oman Animal and Plant Genetic Resource Center. The voucher specimen (DHS-04/2017) was deposited in the herbarium unit at Natural and Medical Sciences Research Center (NMRSC), University of Nizwa, Nizwa, Oman. The provided samples were washed with distilled water (DW) and transported in cool boxes to the laboratory. The cleaned material was freeze dried and ground to make powder, later stored at −20 °C until further analysis.

### 3.3. Extraction, Isolation, and Purification

The freeze-dried samples of D. hoytii (2.3 kg) were finely extracted in 80% methanol (MeOH, 6 L) at room temperature (10 days, 3 times) and evaporated under reduced pressure to yield a semi-solid crude MeOH extract (500 g). The crude MeOH extract was subjected to the silica gel (70–230 mesh; Merck) column chromatography (CC) using n-hexane, n-hexane/ethyl acetate (EtOAc), and EtOAc/MeOH with 10% increasing polarity to afford fourteen fractions (DHF1–DHF14) (Ref.). Fraction DHF1 was loaded over silica gel column chromatography (CC) to acquire pure oil using n-hexane solvent, which was dried over anhydrous sodium sulphate, and kept at 4 °C under subdued light prior to further analysis. Fraction DHF4 (30% n-hexane/EtOAc) were combined and subjected to CC using n-hexane/EtOAc with increasing polarity (1:9, 2:8, 3:7, 4:6, and 5:5) to afford compounds **3** (4.5 mg) and **4** (3.0 mg) along with known compounds **7** (10.0 mg), **9** (12.5 mg), **10** (15.5 mg), **11** (16.0 mg), and **12** (8.0 mg). Similarly, DHF6 (40% n-hexane/EtOAc) was further loaded over silica gel CC to obtain compounds **1** (7.5 mg), **2** (6.0 mg), **5** (8.0 mg), **6** (4.0 mg), and **8** (5.0 mg) using solvent system of 20–40% n-hexane/EtOAc. (Figure S1)

(E)-3-(2-formylphenyl)acrylic acid (**1**): Yellow powder; ^1^HNMR (DMSO-*d*_,_ 600 MHz): δ 10.00 (1H, s), 7.92 (4H, dd, *J* = 7.8, 7.2 Hz), 7.65 (1H, d, *J* = 16.2 Hz), 6.69 (1H, d, *J* = 16.2 Hz); ^13^C-NMR (DMSO-*d*_,_ 125 MHz): δ 192.7, 167.2, 142.4, 139.9, 136.8, 129.9, 128.8, 122.4; HRMS (ESI^+^) *m*/*z*: 214.9809 [M + K]^+^ [38].

Apo-9′-fucoxanthinone (**2**): Colorless amorphous powder; ^1^HNMR (CDCl_3,_ 600 MHz): δ 5.84 (1H, s), 5.39 (1H, m), 2.31 (1H, m), 2.26 (3H, s), 2.16 (3H, s), 1.64 (2H, t), 1.40 (6H, br.s), 1.13 (2H, s); ^13^C-NMR (CDCl_3_, 125 MHz): δ 209.5, 197.9, 170.3, 118.5, 100.9, 72.0, 67.4, 45.1, 45.0, 36.0, 31.6, 30.8, 28.9, 26.4, 21.3; HRMS (ESI^+^) *m*/*z*: 289.1331 [M + Na]^+^; 555.2754 [2M + Na]^+^ [41].

Dimethyl 2-bromoterepthalate (**3**): Amorphous powder; ^1^HNMR (CDCl_3,_ 600 MHz): δ 8.29 (1H, d = 1.8 Hz, H-3), 7.98 (1H, dd = 7.8, 1.8 Hz, H-5), 7.79 (1H, d = 7.8 Hz, H-6), 3.93 (6H, s, OCH_3_); ^13^C-NMR (CDCl_3_, 125 MHz): δ 166.1 (C-7), 164.9 (C-8), 136.1 (C-4), 135.1 (C-3), 133.7 (C-1), 131.0 (C-6), 128.0 (C-5), 121.4 (C-2), 52.7 (C-9), 52.6 (C-10); HRMS (ESI^+^) *m*/*z*: 294.9581 ([^79^BrM + Na]^+^ (Calcd. 294.9582 for C_10_H_9_BrNaO_4_), 296.9560 ([^81^BrM + Na]^+^ (Cald. 296.9562 for C_10_H_9_BrNaO_4_).

Dimethyl 2,6-dibromoterepthalate (**4**): Colorless amorphous powder; ^1^HNMR (CDCl_3,_ 600 MHz): δ 8.04 (2H, s, H-3/6), 3.96 (3H, s, OCH_3_), 3.88 (3H, s, OCH_3_); ^13^C-NMR (CDCl_3_, 125 MHz): δ 166.3 (C-7/8), 133.9 (C-2/5), 129.5 (3/6), 52.4 (C-9/10); HRMS (ESI^+^) *m*/*z*: 367.8938 ([M + H_2_O]^+^ (Calcd. 367.8895 for C_10_H_10_Br_2_O_5_).

(E)-3-(4-(dimethoxymethyl)phenyl)acrylic acid (**5**): Yellow powder; ^1^HNMR (DMSO-*d*_,_ 600 MHz): δ 10.00 (OH), 7.67 (2H, d, *J* = 8.4 Hz, H-3/5), 7.56 (1H, d, *J* = 15.6 Hz, H-8), 7.40 (2H, d, *J* = 8.4 Hz, H-2/6), 6.52 (1H, d, *J* = 15.6 Hz, H-9), 5.38 (1H, s, H-7), 3.23 (6H, 2×OCH_3_); ^13^C-NMR (DMSO-*d*, 125 MHz): δ 167.3 (C-10), 143.0 (C-8), 140.0 (C-4), 134.4 (C-1), 129.9 (C-9), 127.9 (C-3/5), 127.0 (C-2/6), 102.3 (C-7), 52.6 (2×OCH_3_); HRMS (ESI^+^) *m*/*z*: 208. 1723 [M + 2H]^+^; 230.9619 [M + Na]+ (Calcd. 231.0638 for C_11_H_12_NaO_4_).

Pentatetracontanoic acid (**6**): Colorless powder; ^1^HNMR (CDCl_3,_ 600 MHz): δ 2.32 (2H, t, J = 7.8, 7.2 Hz), 1.62 (2H, m), 1.23 (long chain CH_2_), 0.86 (3H, t, J = 6.6Hz); ^13^C-NMR (CDCl_3_, 125 MHz): δ 179.8, 34.1, 31.9, 29.7, 29.6, 29.4, 29.2, 29.1, 24.7, 22.7, 14.1; HRMS (ESI^+^) *m*/*z*: 663.4575 [M]^+^, 647.4626 [M − OH + H] [42,43].

Octadec-1-ene (**7**): Colorless amorphous powder; ^1^HNMR (CDCl_3,_ 600 MHz): δ 5.82 (1H, m), 4.98 (1H, d, J = 16.8 Hz), 4.91 (1H, d, J = 10.2 Hz), 2.03 (2H, dd, J = 7.2, 6.6 Hz), 1.23 (long chain CH_2_, br.s), 0.87 (3H, t, J = 6.6 Hz); ^13^C-NMR (CDCl_3_, 125 MHz): δ 139.3, 114.1, 33.8, 31.9, 29.7, 29.6, 29.5, 29.4, 29.0, 22.7, 14.1; HRMS (ESI^+^) *m*/*z*: 275.2356 [M + Na]^+^ [44].

Epi-amyrine (**8**): Colorless powder; ^1^HNMR (CDCl_3,_ 600 MHz): δ 5.11 (1H, t, J = 3.5, H-12), 3.38 (1H, br s, H-3), 0.78–0.98 (21H, m, 7 × CH_3_) and 1.06 (3H, s, CH_3_); ^13^C-NMR (CDCl_3_, 125 MHz): δ 139.5, 124.4, 76.8, 59.0, 48.9, 47.5, 42.1, 40.1, 39.6, 39.5, 37.3, 36.9, 33.7, 33.2, 32.8, 31.2, 28.7, 28.2, 28.1, 26.5, 21.4, 18.4, 17.5, 16.9, 15.5; HRMS (ESI^+^) *m*/*z*: 426.9673 [M + H]^+^ [45,46].

1,4-Benzenedicarboxaldehyde (**9**): ^1^HNMR (DMSO-*d*_,_ 600 MHz): δ 9.80 (1H, s), 7.60 (2H, d, J = 7.8 Hz), 7.30 (2H, d, J = 7.8 Hz); ^13^C-NMR (DMSO-*d*_,_ 125 MHz): δ 192.6, 134.1, 128.9, 128.8 [47,48].

Tricosylic acid (**10**): ^1^HNMR (CDCl_3,_ 600 MHz): δ 2.28 (2H, t, J = 7.8, 7.2 Hz), 1.59 (2H, t, J = 7.2 Hz), 1.29 (long chain CH_2_), 0.85 (3H, t, 7.2, 6.6 Hz); ^13^C-NMR (CDCl_3_, 125 MHz): δ 176.7, 33.9, 31.9, 29.7, 29.6, 29.4, 29.3, 29.1, 24.8, 22.7, 14.1; HRMS (ESI^+^) *m*/*z*: 377.2004 [M + Na]^+^ [49].

Hexadecanoic acid (**11**): ^1^HNMR (CDCl_3,_ 600 MHz): δ 2.30 (2H, t, J = 7.8 Hz), 1.61 (2H, m), 1.29 (long chain CH_2_), 0.86 (3H, t, J = 6.6 Hz); ^13^C-NMR (CDCl_3_, 125 MHz): δ 177.6, 34.0, 31.9, 29.7, 29.6, 29.4, 29.3, 29.1, 24.8, 22.7, 14.1; HRMS (ESI^−^) *m*/*z*: 255.2335 [M − H]^+^ [49,50].

Lacceroic acid (**12**): ^1^HNMR (CDCl_3,_ 600 MHz): δ 2.30 (2H, t, J = 7.8, 7.2 Hz), 1.61 (2H, m), 1.30 (long chain CH_2_), 0.86 (3H, t = 7.2, 6.6 Hz); ); ^13^C-NMR (CDCl_3_, 125 MHz): δ 177.5, 33.9, 31.9, 29.7, 29.6, 29.4, 29.3, 29.1, 24.8, 22.7, 14.1; HRMS (ESI^+^) *m*/*z*: 481.2651 [M]^+^ [51,52].

### 3.4. Carbonic Anhydrase II Inhibition

The total reaction volume of 200 µL including 20 µL of test compounds, isolated from *Dictyopteris hoytii*, and prepared in DMSO, followed by the addition of 140 µL of HEPES–tris buffer, 20 µL of purified bovine erythrocyte CA-II (0.1 mg/mL) prepared in buffer, and 20 µL of a solution of 4-nitrophenyl acetate [53]. Then, 20 µL of the test compound was incubated with the enzyme carbonic anhydrase (EC 4.2.1.1) for 15 min in 96-well flat bottom plates. The rate of product formation was monitored with the addition of 20 µL of 4-NPA as substrate, prepared in methanol at the final concentration of 0.7 mM, at 25 °C for 30 min with regular intervals of 1 min, by using microplate readers (Bio-Rad, Molecular Devices, CA, USA). HEPES-tris was used as a buffer for the reaction at a final concentration of 20 mM at pH 7.4. Acetazolamide was used as standard inhibitor (IC_50_ = 18.2 ± 1.23 µM).

### 3.5. Molecular Docking and ADMET Prediction

The docking was performed by MOE docking program [54]. The human CA-II structure in complex with ligand (acetazolamide derivative) was chosen from Protein Databank (PDB code 4IWZ [55], resolution = 1.60Å). The 3D structure of protein was prepared for docking by removing heteroatoms other than the co-crystallized ligand, ZN ion, and the water molecules within the 3Å region of the active site. Hydrogen atoms were added on protein structure and partial charges were applied using MMFF94x force field through MOE Protonate 3D option. The modified protein file was saved in PDB format. The 2D-structures of compounds **1**, **3**, **5**, **7**, **9**–**10**, **12**–**13**, **15**, **18**, and **19** were sketched on ChemDraw Ultra 8.0 and imported into MOE database in which structures were converted into 3D-form by applying minimization (RMS Gradient = 0.1 Kcal/mol/Å). Partial charges (MMFF94x) and hydrogen atoms were added during minimization automatically. After preparation of protein and ligand files, docking was performed with Induced Fit docking protocol of MOE using the Triangle Matcher docking algorithm and London dG scoring function. Induced Fit protocol is appropriate when protein sidechains are to be included in the refinement stage. The co-crystallized ligand and acetazolamide were added in the ligand database as positive controls. The co-crystallized ligand adapts similar docked conformation like in the X-ray crystal structure and showed almost similar binding pattern after docking with RMSD value of 0.96Å (Figure S2). Afterwards, the physicochemical and pharmacokinetic properties (absorption, distribution, metabolism, excretion, and toxicity) of the compounds were predicted through Swiss ADME server (http://www.swissadme.ch, accessed on 1 August 2021)

## 4. Conclusions

Twelve compounds (**1**–**12**) were isolated from the methanolic extract of *D. hoytii* through column chromatography. Compounds **3–5** were identified as natural products for the first time in this study while rest of the compounds (**1**, **2**, **6–12**) were known, and were isolated for the first time from the natural source. Among the tested compounds, compounds **4**, **5**, **7**, **12**, and **18** were identified as having the most potential hits and may be excellent inhibitors against CA-II. These natural products can eventually be used as lead scaffolds for future drug discovery design and development against cancer. Furthermore, the results of the current investigation also suggest that the selected marine seaweed may serve as a source of novel bioactive natural products and provide a basis to search an alternative source of therapeutic agents.

## Figures and Tables

**Table 1:** The carbonic anhydrase-II inhibitory activities of the isolated compounds.

Compounds	% Inhibition	IC_50_ ± SEM
**1**	79.4	44.9 ± 1.10
**2**	17.9	NA
**3**	16.6	NA
**4**	72.8	13.4 ± 1.35
**5**	93.9	17.7 ± 2.01
**6**	ND	ND
**7**	88.0	16.3 ± 0.31
**8**	ND	ND
**9**	93.9	53.1 ± 1.20
**10**	77.7	71.6 ± 1.05
**11**	20.7	NA
**12**	90.3	11.6 ± 0.56
**13**	87.3	39.5 ± 0.73
**14**	ND	ND
**15**	69.8	31.3 ± 1.34
**16**	37.4	NA
**17**	ND	ND
**18**	77.5	17.0 ± 1.32
**19**	87.3	24.3 ±1.38
**20**	29.5	NA
Acetazolamide	86.4	18.2 ± 1.23

NA = not active; ND = not determined; SEM =standard error mean.

**Table 2:** The docking scores and binding interactions of compounds **1**, **4**, **5**, **7**, **9–10**, **12–13**, **15**, **18–19**, and acetazolamide.

Compounds.	IC_50_ ± S.E.M	Docking Score (kcal/mol)	Binding Interactions			
			Ligand	Receptor	Bonds	Distance (Å)
**12**	11.6 ± 0.56	−9.53	O93	NE2-HIS94	HBA	3.15
			O94	N-THR199	HBA	3.06
			O94	OG1-THR199	HBA	3.05
			O94	OG1-THR200	HBA	3.45
			O93	ZN	Ionic	2.37
			O93	ZN	Ionic	2.37
			O94	ZN	Ionic	3.15
**4**	13.4 ± 1.35	−5.06	O14	NE2-HIS94	HBA	3.15
			O14	ZN	Ionic	2.38
**7**	16.3 ± 0.31	−9.00	C8	5-ring-HIS94	H-π	3.13
**18**	17.0 ± 1.32	−9.35	O78	NE2-HIS94	HBA	2.83
			O79	N-THR199	HBA	3.03
			O78	ZN	Ionic	2.30
			O78	ZN	Ionic	2.30
**5**	17.7 ± 2.01	−5.39	O18	N-THR199	HBA	3.15
			O18	OG1-THR199	HBA	3.44
			O19	NE2-HIS94	HBA	2.89
			O19	NE2-HIS96	HBA	3.36
			O19	ZN	Ionic	2.27
			O19	ZN	Ionic	2.27
**19**	24.3 ±1.38	−9.66	O83	N-THR199	HBA	3.03
			O83	OG1-THR199	HBA	2.90
			O82	ZN	Ionic	2.42
			O83	ZN	Ionic	2.67
			O82	ZN	Ionic	2.42
			O83	ZN	Ionic	2.67
**15**	31.3 ± 1.34	−5.19	O61	NE2-HIS94	HBD	2.81
			O61	ZN	Ionic	2.52
**13**	39.5 ± 0.73	−5.83	O14	NE2-HIS94	HBA	3.15
			O14	ZN	Ionic	2.55
**1**	44.9 ± 1.10	−5.70	O19	N-THR199	HBA	3.38
			O19	OG1-THR199	HBA	2.90
			O13	ZN	Ionic	2.88
			O19	ZN	Ionic	2.34
			O19	ZN	Ionic	2.34
**9**	53.1 ± 1.20	−4.58	O13	OG1-THR199	HBA	3.02
			O13	ZN	Ionic	2.49
**10**	71.6 ± 1.05	−9.35	O66	NE2-HIS94	HBA	2.93
			O67	N-THR199	HBA	3.05
			O66	ZN	Ionic	2.26
			O66	ZN	Ionic	2.26
Acetazolamide	18.2 ± 1.23	−9.47	N1	NE2-HIS94	HBA	2.89
			N1	NE2-HIS96	HBA	3.32
			N1	OG1-THR199	HBA	3.28
			O1	N-THR199	HBA	2.90
			O1	OG1-THR199	HBA	3.54
			N1	ZN	Ionic	1.82
			N1	ZN	Ionic	1.82
			O2	ZN	Ionic	3.33
			5-ring	CD2-LEU198	π-H	3.75

## Data Availability

The data presented in this study are available in Supplementary Material.

## Supplementary Materials

The following are available online, Figure S1: ^1^H, ^13^C NMR, and HR-ESI-MS of the compounds **1**–**12**; Figure S2: Superimposed view of co-crystalized ligand in 4IZW. The docked and the X-ray conformations of compound are shown in gray and green colors, respectively; Table S1: The predicted ADMET properties of compounds.

## Sample Availability

Samples of the compounds are not available from the authors.

## References

[1] Lourenço-Lopes C., Fraga-Corral M., Jimenez-Lopez C., Pereira A.G., Garcia-Oliveira P., Carpena M., Prieto M.A., Simal-Gandara J. (2020). Metabolites from Macroalgae and Its Applications in the Cosmetic Industry: A Circular Economy Approach. Resources.

[2] Leandro A., Pereira L., Gonçalves A.M.M. (2020). Diverse Applications of Marine Macroalgae. Mar. Drugs.

[3] Biris-Dorhoi E.-S., Michiu D., Pop C.R., Rotar A.M., Tofana M., Pop O.L., Socaci S.A., Farcas A.C. (2020). Macroalgae—A Sustainable Source of Chemical Compounds with Biological Activities. Nutrients.

[4] Silva A., Silva S.A., Carpena M., Garcia-Oliveira P., Gullón P., Barroso M.F., Prieto M.A., Simal-Gandara J. (2020). Macroalgae as a Source of Valuable Antimicrobial Compounds: Extraction and Applications. Antibiotics.

[5] Melendres A.R., Ilano A. (2017). Bio-oil Product from Wild Brown Macro-algae Dunggandunggan (Padinasp) in Asturias and Carmen, Cebu, Philippines. Int. J. Med. Plants Nat. Prod..

[6] Rajasulochana A., Dhamotharan R., Krishnamoorthy P., Subbiah M. (2009). Antibacterial Activity of the Extracts of Marine Red and Brown. J. Am. Sci..

[7] Leal M.C., Munro M.H.G., Blunt J.W., Puga J., Jesus B., Calado R., Rosa R., Madeira C. (2013). Biogeography and biodiscovery hotspots of macroalgal marine natural products. Nat. Prod. Rep..

[8] Carroll A.R., Copp B.R., Davis R.A., Keyzers R.A., Prinsep M.R. (2020). Marine natural products. Nat. Prod. Rep..

[9] Moore R.E., Pettus J.A. (1971). Isolation and structure determination of dictyopterenes C’ and D’ from Dictyopteris. Stereospecificity in the cope rearrangement of dictyopterenes A and B. J. Am. Chem. Soc..

[10] Schnitzler I., Pohnert G., Hay M., Boland W. (2001). Chemical defense of brown algae (*Dictyopteris* spp.) against the herbivorous amphipod *Ampithoe longimana*. Oecologia.

[11] Zatelli G.A., Philippus A.C., Falkenberg M. (2018). An overview of odoriferous marine seaweeds of the Dictyopteris genus: Insights into their chemical diversity, biological potential and ecological roles. Rev. Bras. Farmacogn..

[12] Feng M.-T., Wang T., Liu A.-H., Li J., Yao L.-G., Wang B., Guo Y.-W., Mao S.-C. (2018). PTP1B inhibitory and cytotoxic C-24 epimers of Δ28-24-hydroxy stigmastane-type steroids from the brown alga Dictyopteris undulata Holmes. Phytochemistry.

[13] Ji N.-Y., Wen W., Li X.-M., Xue Q.-Z., Xiao H.-L., Wang B.-G. (2009). Brominated Selinane Sesquiterpenes from the Marine Brown Alga Dictyopteris divaricata. Mar. Drugs.

[14] Song F., Xu X., Li S., Wang S., Zhao J., Yang Y., Fan X., Shi J., He L. (2006). Minor sesquiterpenes with new carbon skeletons from the brown alga Dictyopteris divaricata. J. Nat. Prod..

[15] Rehman N.U., Rafiq K., Khan A., Halim S.A., Ali L., Al-Saady N., Al-Balushi A.H., Al-Busaidi H.K., Al-Harrasi A. (2019). α-Glucosidase inhibition and molecular docking studies of natural brominated metabolites from marine macro brown alga Dictyopteris hoytii. Mar. Drugs.

[16] Segawa M., Yamano K., Shirahama H. (1990). A germacrane-type sesquiterpene from the brown alga Dictyopteris divaricata. Phytochemistry.

[17] Zbakh H., Zubía E., De Los Reyes C., Calderón-Montaño J.M., Motilva V. (2020). Anticancer Activities of Meroterpenoids Isolated from the Brown Alga Cystoseira usneoides against the Human Colon Cancer Cells HT-29. Foods.

[18] Song F., Fan X., Xu X., Zhao J., Yang Y., Shi J. (2004). Cadinane sesquiterpenes from the brown alga Dictyopteris divaricata. J. Nat. Prod..

[19] Kurata K., Taniguchi K., Suzuki M. (1996). Cyclozonarone, a sesquiterpene-substituted benzoquinone derivative from the brown alga Dictyopteris undulata. Phytochemistry.

[20] Song F., Xu X., Li S., Wang S., Zhao J., Cao P., Yang Y., Fan X., Shi J., He L. (2005). Norsesquiterpenes from the brown alga Dictyopteris divaricata. J. Nat. Prod..

[21] Moore R.E., Pettus J.A., Mistysyn J. (1974). Odoriferous C11 hydrocarbons from Hawaiian Dictyopteris. J. Org. Chem..

[22] Dimou M., Ioannou E., Daskalaki M.G., Tziveleka L.A., Kampranis S.C., Roussis V. (2016). Disulfides with Anti-inflammatory Activity from the Brown Alga Dictyopteris membranacea. J. Nat. Prod..

[23] Shimizu H., Koyama T., Yamada S., Lipton S.A., Satoh T. (2015). Zonarol, a sesquiterpene from the brown algae Dictyopteris undulata, provides neuroprotection by activating the Nrf2/ARE pathway. Biochem. Biophys. Res. Commun..

[24] Belattmania Z., Reani A., Mustapha B., Zrid R., Samir E.A., Hassouani M., Eddaoui A., Bentiss F., Sabour B. (2016). Antimicrobial, antioxidant and alginate potentials of Dictyopteris polypodioides (Dictyotales, Phaeophyceae) from the Moroccan Atlantic coast. Der Pharma Chem..

[25] Kim K.N., Ham Y.M., Moon J.Y., Kim M.J., Kim D.S., Lee W.J., Lee N.H., Hyun C.G. (2009). In vitro cytotoxic activity of Sargassum thunbergii and Dictyopteris divaricata (Jeju seaweeds) on the HL-60 tumour cell line. Int. J. Pharmacol..

[26] Ghorai S., Pulya S., Ghosh K., Panda P., Ghosh B., Gayen S. (2020). Structure-activity relationship of human carbonic anhydrase-II inhibitors: Detailed insight for future development as anti-glaucoma agents. Bioorg. Chem..

[27] Achal V., Pan X. (2010). Characterization of Urease and Carbonic Anhydrase Producing Bacteria and Their Role in Calcite Precipitation. Curr. Microbiol..

[28] Karakaya S., Bingol Z., Koca M., Dagoglu S., Pınar N.M., Demirci B., Gulcin İ., Brestic M., Sytar O. (2020). Identification of non-alkaloid natural compounds of Angelica purpurascens (Avé-Lall.) Gilli. (Apiaceae) with cholinesterase and carbonic anhydrase inhibition potential. Saudi Pharm. J..

[29] Masini E., Carta F., Scozzafava A., Supuran C.T. (2013). Antiglaucoma carbonic anhydrase inhibitors: A patent review. Expert Opin. Ther. Pat..

[30] Costa G., Gidaro M.C., Vullo D., Supuran C.T., Alcaro S. (2016). Active Components of Essential Oils as Anti-Obesity Potential Drugs Investigated by in Silico Techniques. J. Agric. Food Chem..

[31] Muhammad N., Saeed M., Adhikari A., Khan K.M., Khan H. (2013). Isolation of a new bioactive cinnamic acid derivative from the whole plant of Viola betonicifolia. J. Enzyme Inhib. Med. Chem..

[32] Nauroozi D., Pejic M., Schwartz P.-O., Wachtler M., Bäuerle P. (2016). Synthesis and solvent-free polymerisation of vinyl terephthalate for application as an anode material in organic batteries. RSC Adv..

[33] Katz M.J., Brown Z.J., Colón Y.J., Siu P.W., Scheidt K.A., Snurr R.Q., Hupp J.T., Farha O.K. (2013). A facile synthesis of UiO-66{,} UiO-67 and their derivatives. Chem. Commun..

[34] Song C.L., Liu K., Zhang A.J., Xu Z.G., Zhang H.L. (2011). Dimethyl 2,5-bis-(5-hexyl-thio-phen-2-yl)benzene-1,4-dioate. Acta Crystallogr. Sect. E Struct. Rep. Online.

[35] Rech J.J., Bauer N., Dirkes D., Kaplan J., Peng Z., Zhang H., Ye L., Liu S., Gao F., Ade H. (2019). The crucial role of end group planarity for fused-ring electron acceptors in organic solar cells. Mater. Chem. Front..

[36] Daluge S.M., Wolberg G., Livingston D.A., SmithKline Beecham Corp (1998). Substituted (1,3-bis(cyclohexylmethyl)-1,2,3,6-tetrahydro-2,6-dioxo-9h-purin-8-yl) Phenyl Derivatives, Their Preparation and Their Use in the Treatment of Inflammatory Conditions and Immune Disorders. US Patent.

[37] Lehmann-Lintz T., Lustenberger P., Jürgen Roth G., Schindler M., Thomas L., Georg Mueller S., Stenkamp D., Lotz R.R.H., Rudolf K. (2005). 3-(4-piperidine-1ylmethyl-phenyl)-propion Acid-phenylamide-derivatives and Related Compounds Used in the Form of mch Antagonists (Melanine Concentrating Hormone) for Treating Eating Disorders. US Patent.

[38] Bernatek E., Frengen C. (1962). Ozonolysis of Phenols. III. 1- and 2-Naphthol. Acta Chem. Scand..

[39] Chatterjee B.G., Moza P.N. (1966). Synthesis of Substituted β-Lactams. J. Med. Chem..

[40] Hattab M., Culioli G., Valls R., Richou M., Piovetti L. (2008). Apo-fucoxanthinoids and loliolide from the brown alga Cladostephus spongiosus f. verticillatus (Heterokonta, Sphacelariales). Biochem. Syst. Ecol..

[41] Doi Y., Ishibashi M., Yamaguchi N., Kobayashi J. (1995). Isolation of Apo-9’-fucoxanthinone from the Cultured Marine Dinoflagellate Amphidinium sp.. J. Nat. Prod..

[42] Fengzhi R., Huihua Q., Xinhui L., Yimin Z. (2001). Studies on the chemical constituents of Callicarpa bodinieri Levl. Nat. Prod. Res. Dev..

[43] Dailey O.D., Severson R.F., Arrendale R.F. (1997). Nonpolar Lipids of Amaranthus palmeri S. Wats. 2. Unsaturated Esters and Free Fatty Acids, Sterols, and Triterpenols. J. Agric. Food Chem..

[44] Sulaimon L., Anise E.O., Obuotor E.M., Samuel T., Moshood A., Olajide M., Fatoke T. (2020). In vitro antidiabetic potentials, antioxidant activities and phytochemical profile of african black pepper (Piper guineense). Clin. Phytoscience.

[45] Dekebo A., Dagne E., Aasen O.R.G., Aasen A.J. (2002). Triterpenes from the resin of Boswellia neglecta. Bull. Chem. Soc. Ethiop..

[46] Ur Rehman N., Halim S.A., Al-Azri M., Khan M., Khan A., Rafiq K., Al-Rawahi A., Csuk R., Al-Harrasi A. (2020). Triterpenic Acids as Non-Competitive α-Glucosidase Inhibitors from Boswellia elongata with Structure-Activity Relationship: In Vitro and In Silico Studies. Biomolecules.

[47] Sulzer P., Hartungen E., Hanel G., Feil S., Winkler K., Mutschlechner P., Haidacher S., Schottkowsky R., Gunsch D., Seehauser H. (2014). A Proton Transfer Reaction-Quadrupole interface Time-Of-Flight Mass Spectrometer (PTR-QiTOF): High speed due to extreme sensitivity. Int. J. Mass Spectrom..

[48] Dong J., Xu F., Dong Z., Zhao Y., Yan Y., Jin H., Li Y. (2018). Fabrication of two dual-functionalized covalent organic polymers through heterostructural mixed linkers and their use as cationic dye adsorbents. RSC Adv..

[49] Liu Z., Zhao R., Zou Z. (2011). Chemical constituents from root bark of Tripterygium hypoglaucum. China J. Chin. Mater. Med..

[50] Krishnan K.R., James F., Mohan A. (2016). Isolation and characterization of n-hexadecanoic acid from *Canthium parviflorum* leaves. J. Chem. Pharm. Res..

[51] Mir M. (2019). Isolation, Characterization and Bioactivities of Sambucus wightiana derived Dotriacontanoic acid. Int. J. Trend Sci. Res. Dev..

[52] KALIMUTHU S., Latha S., Selvamani P., Pandiyan R., BALAMURUGAN B., CHANDRASEKAR T.M. (2011). Isolation, Characterization and Antibacterial Evaluation on Long Chain Fatty Acids from Limnophila polystachya Benth. Asian J. Chem..

[53] Innocenti A., Sarıkaya S.B.O., Gülçin İ., Supuran C.T. (2010). Carbonic anhydrase inhibitors. Inhibition of mammalian isoforms I–XIV with a series of natural product polyphenols and phenolic acids. Bioorg. Med. Chem..

[54] Chemical Computing Group Inc. (2014). Molecular Operating Environment (MOE).

[55] Biswas S., McKenna R., Supuran C.T. (2013). Effect of incorporating a thiophene tail in the scaffold of acetazolamide on the inhibition of human carbonic anhydrase isoforms I., II, IX and XII. Bioorg. Med. Chem. Lett..

