# Supplementary material for: New Carbonic Anhydrase-II Inhibitors from Marine Macro Brown Alga Dictyopteris hoytii Supported by In Silico Studies
Source: Molecules. 2021 Nov 23;26(23):7074. doi: 10.3390/molecules26237074 (PMC8658806; doi:10.3390/molecules26237074)
Supplement: Supplementary file 1 [file molecules-26-07074-s001.zip › molecules-1465620-supplementary.pdf]

molecules

Supporting information

New Carbonic Anhydrase-II Inhibitors from Marine Macro Brown Alga *Dictyopteris hoytii* Supported by In Silico Studies

Kashif Rafiq [1,†], Ajmal Khan [1,†], Najeeb Ur Rehman [1,*], Sobia Ahsan Halim [1], Majid Khan [1,2], Liaqat Ali [1,3], Abdullah Hilal Al-Balushi [4], Haitham Khamis Al-Busaidi [4] and Ahmed Al-Harrasi [1,*]

[1] Natural & Medical Sciences Research Center, University of Nizwa, P.O. Box 33, Postal Code 616, Birkat Al Mauz, Nizwa, Oman; kashifrafiq609@gmail.com (K.R.); ajmalkhan@unizwa.edu.om (A.K.); sobia_halim@unizwa.edu.om (S.A.H.); majidk166@yahoo.com (M.K.); malikhejric@gmail.com (L.A.)

[2] H. E. J. Research Institute of Chemistry, International Center for Chemical and Biological Sciences, University of Karachi, Karachi 75270, Pakistan

[3] Department of Chemistry, University of Mianwali, Mianwali 42200, Pakistan

[4] Oman Animal and Plant Genetic Resources Center, P.O. Box 92, 123 Muscat, Oman; abdullah.albalushi@oapgrc.gov.om (A.H.A.-B.); Haihtam.AlBusaidi@oapgrc.gov.om (H.K.A.-B.)

* Correspondence: najeeb@unizwa.edu.om (N.U.R.); aharrasi@unizwa.edu.om (A.A.-H.); Tel.: +968-25446328 (A.A.-H.); Fax: +968-2544-6612 (A.A.-H.)

† These authors contributed equally to this work.

Figure S1: ^{1}H, ^{13}C NMR, and HR-ESI-MS of the compounds **1-12**

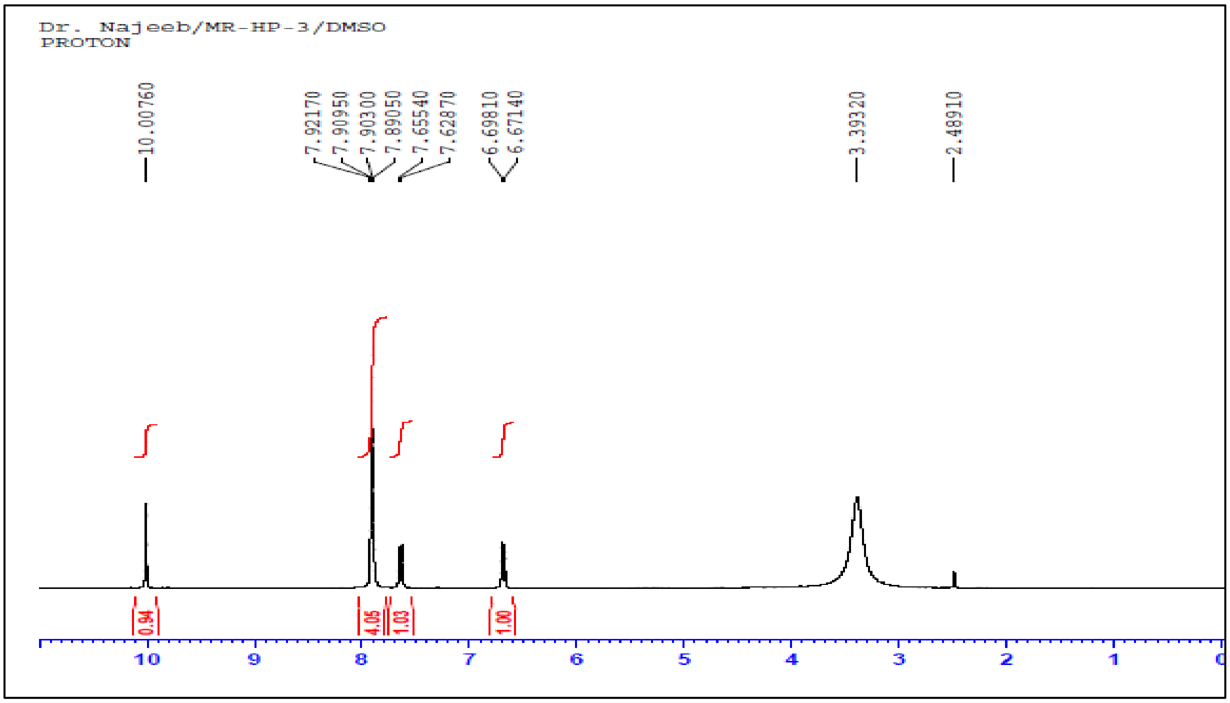

^{1}H NMR (600 MHz) of compound **1** DMSO-*d*

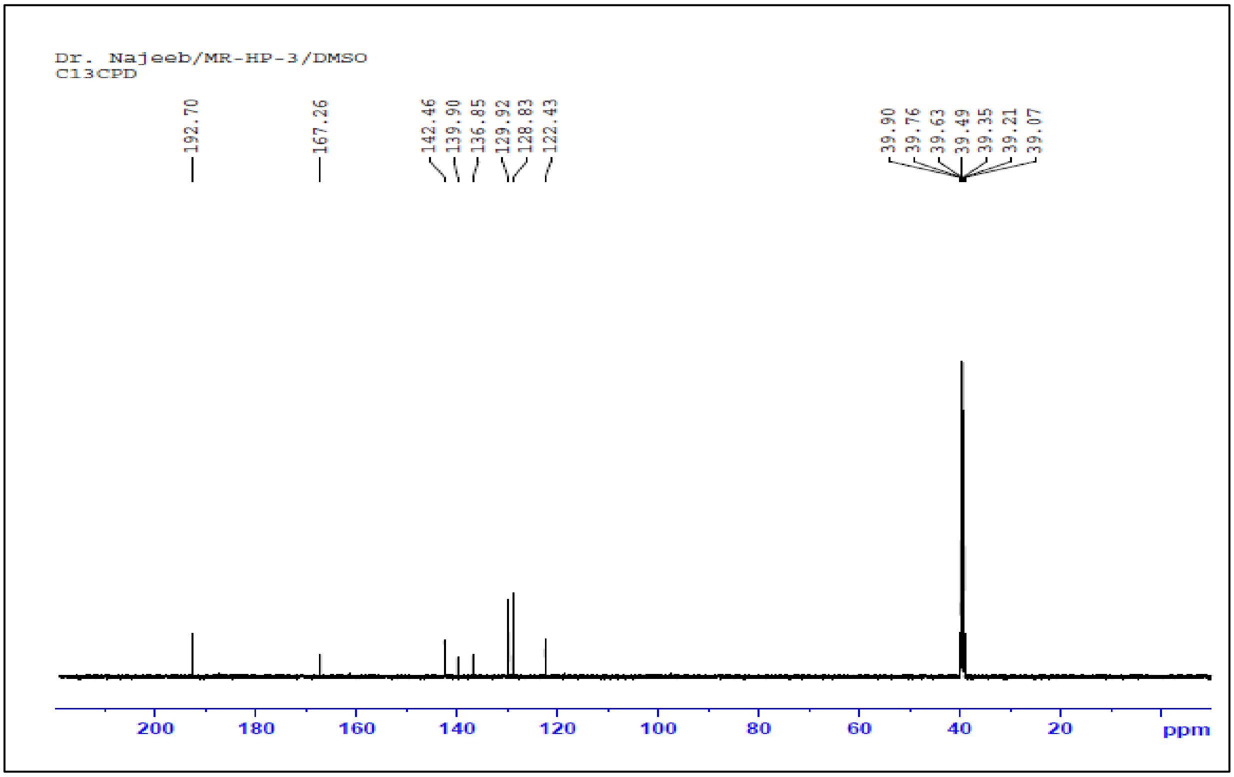

^{13}C NMR (125 MHz) of compound **1** in DMSO-*d*

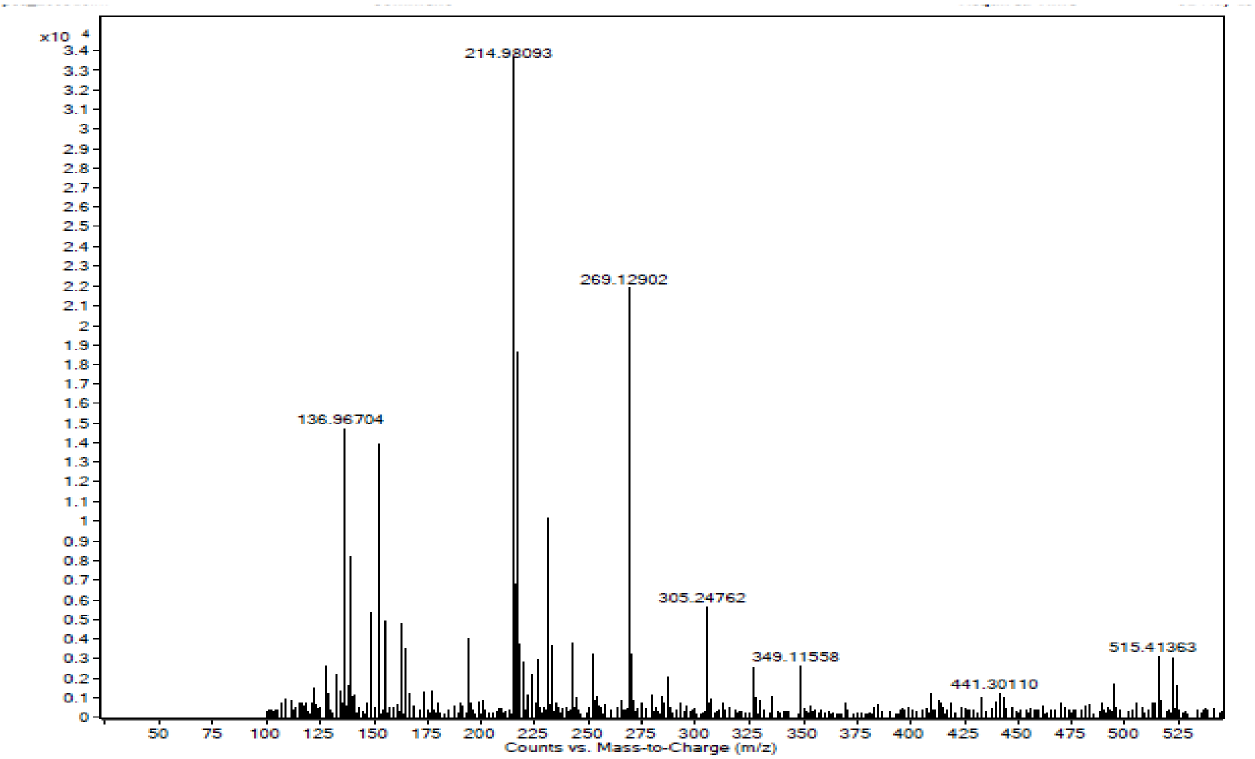

HR-ESI-MS (positive) of compound **1**

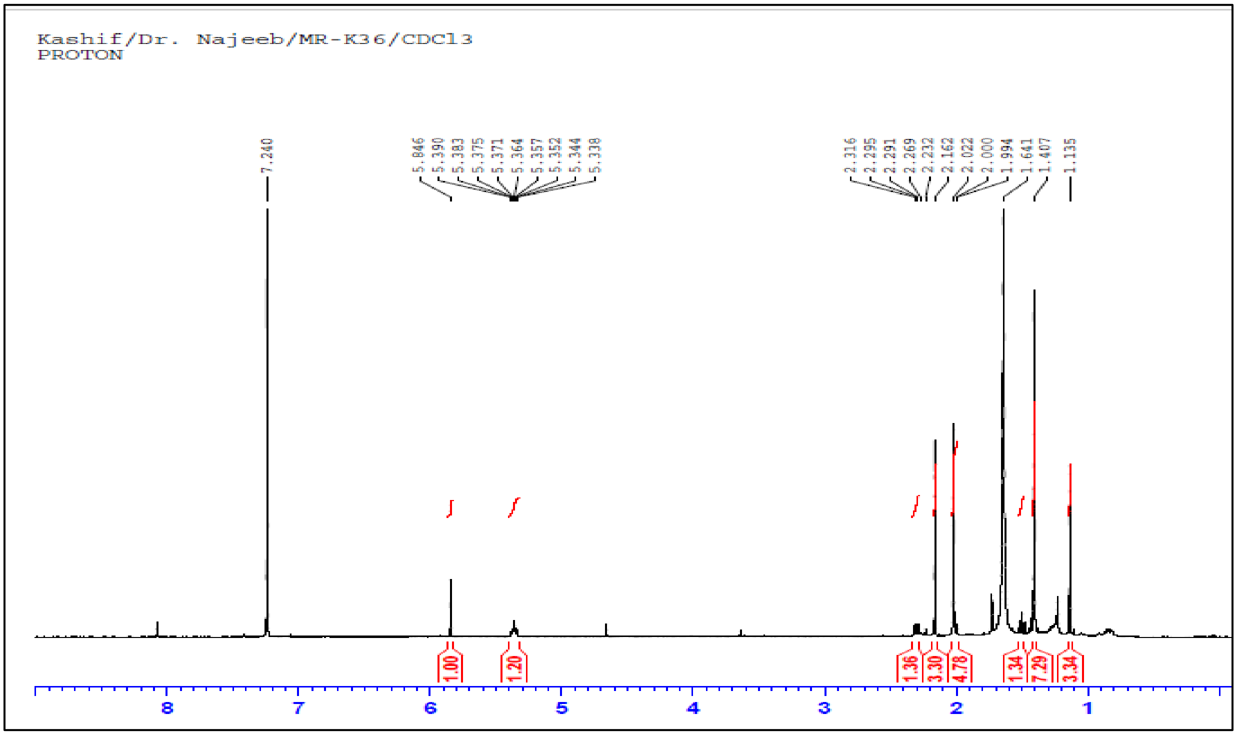

^{1}H NMR (600 MHz) of compound **2** in $CDCl_3$

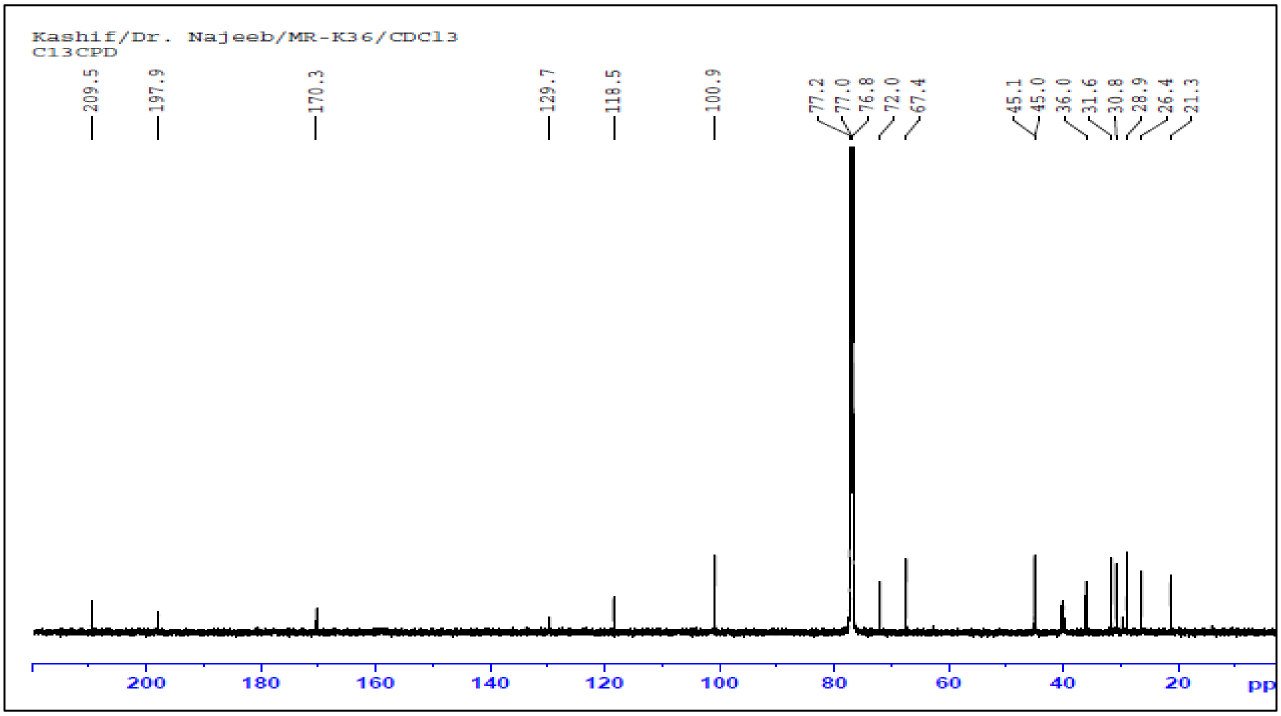

^{13}C NMR (125 MHz) of compound **2** in $CDCl_3$

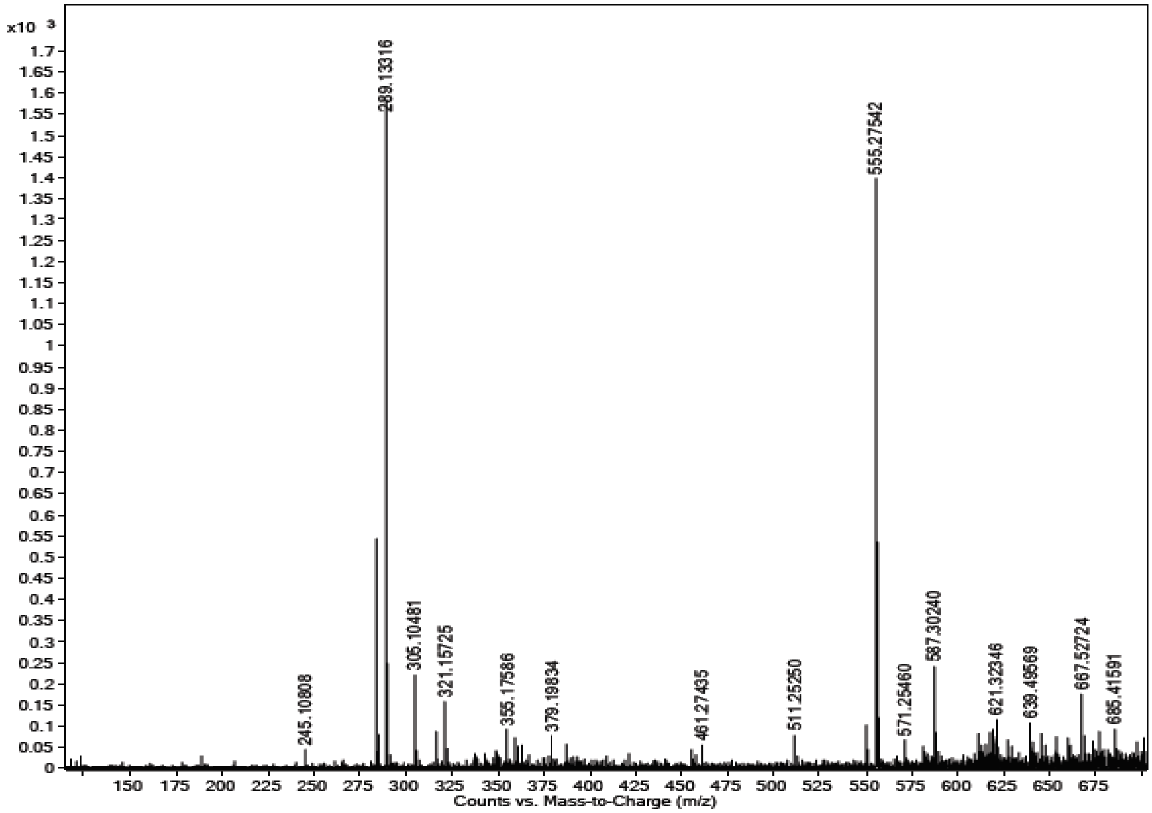

HR-ESI-MS (positive) of compound **2**

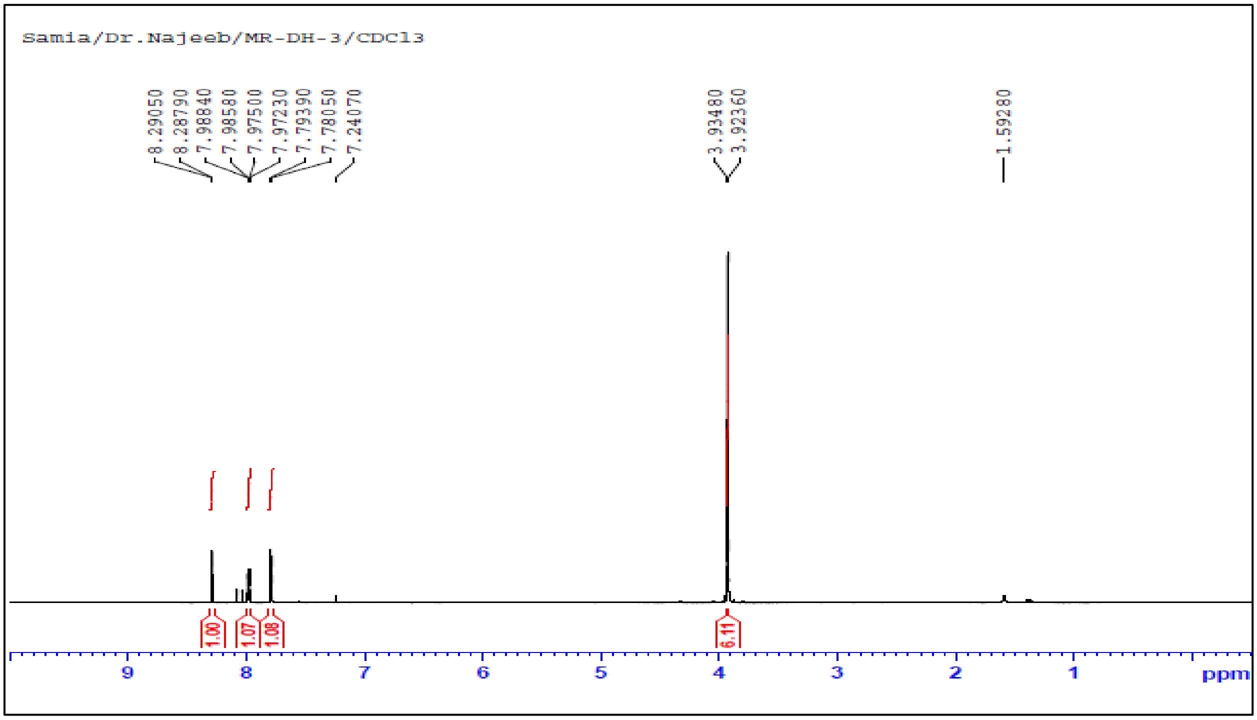

^1H NMR (600 MHz) of compound **3** in $CDCl_3$

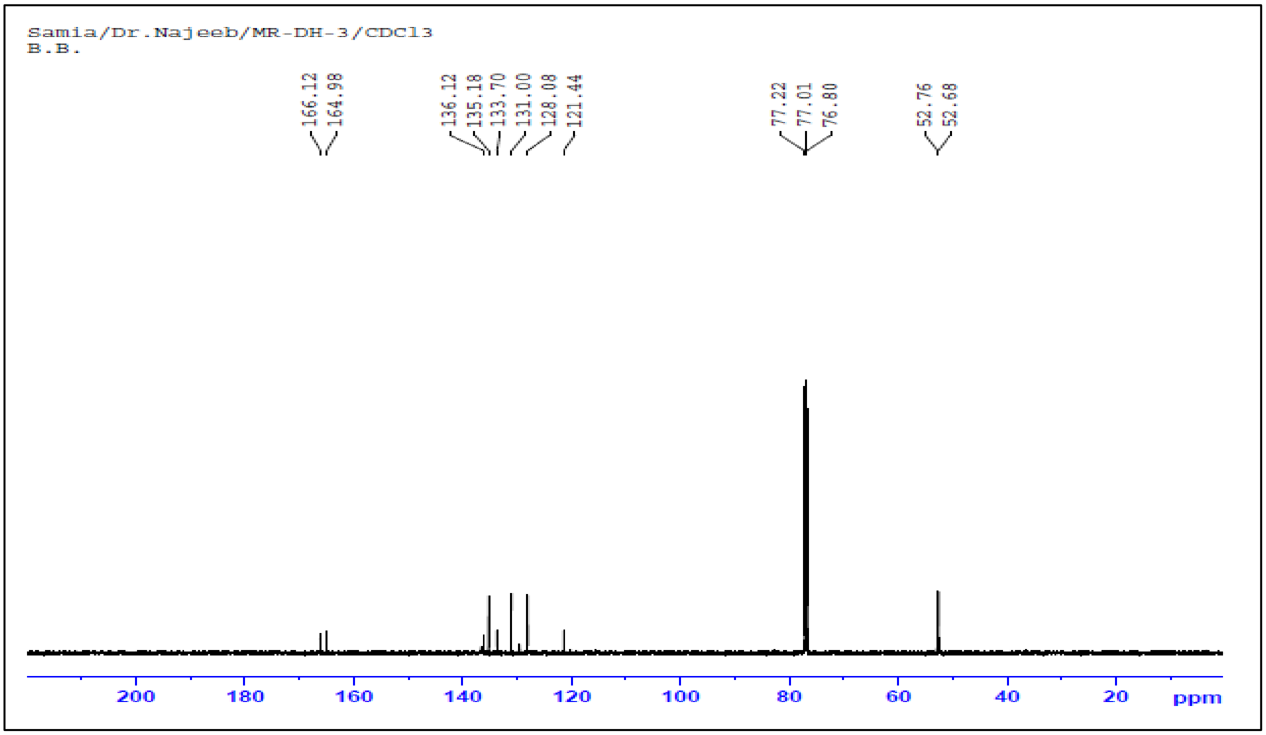

^{13}C NMR (125 MHz) of compound **3** in $CDCl_3$

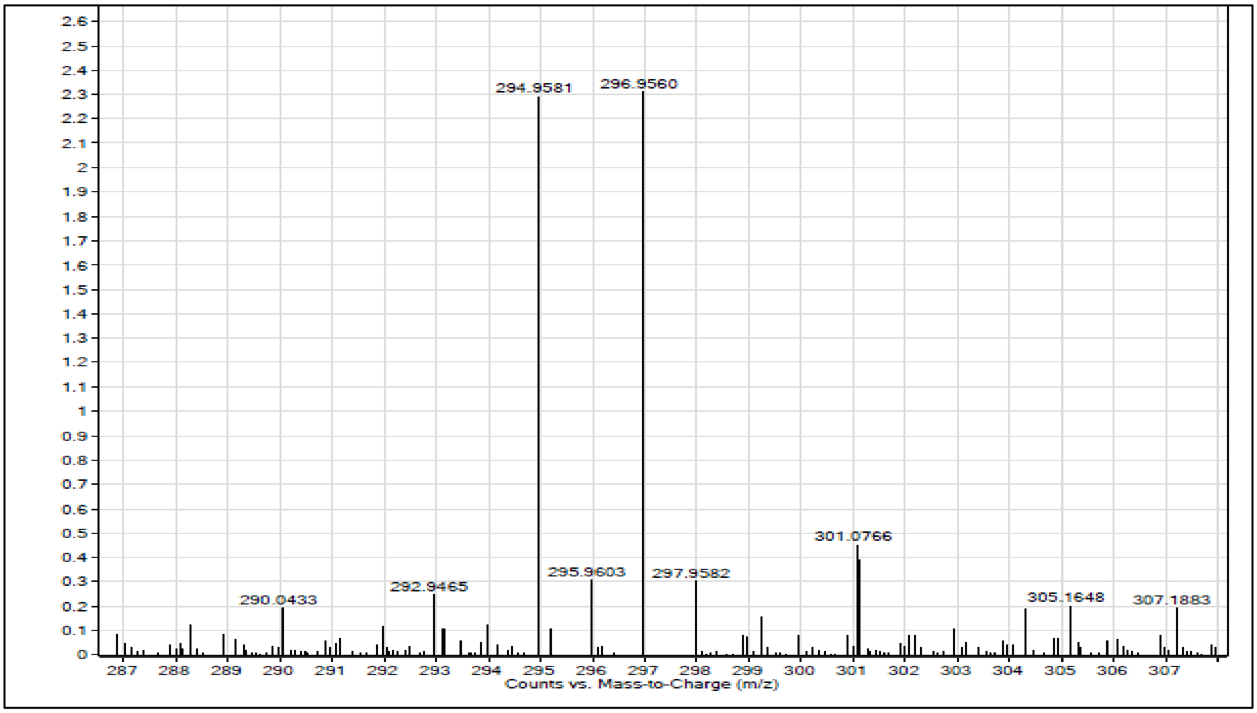

HR-ESI-MS (positive) of compound **3**

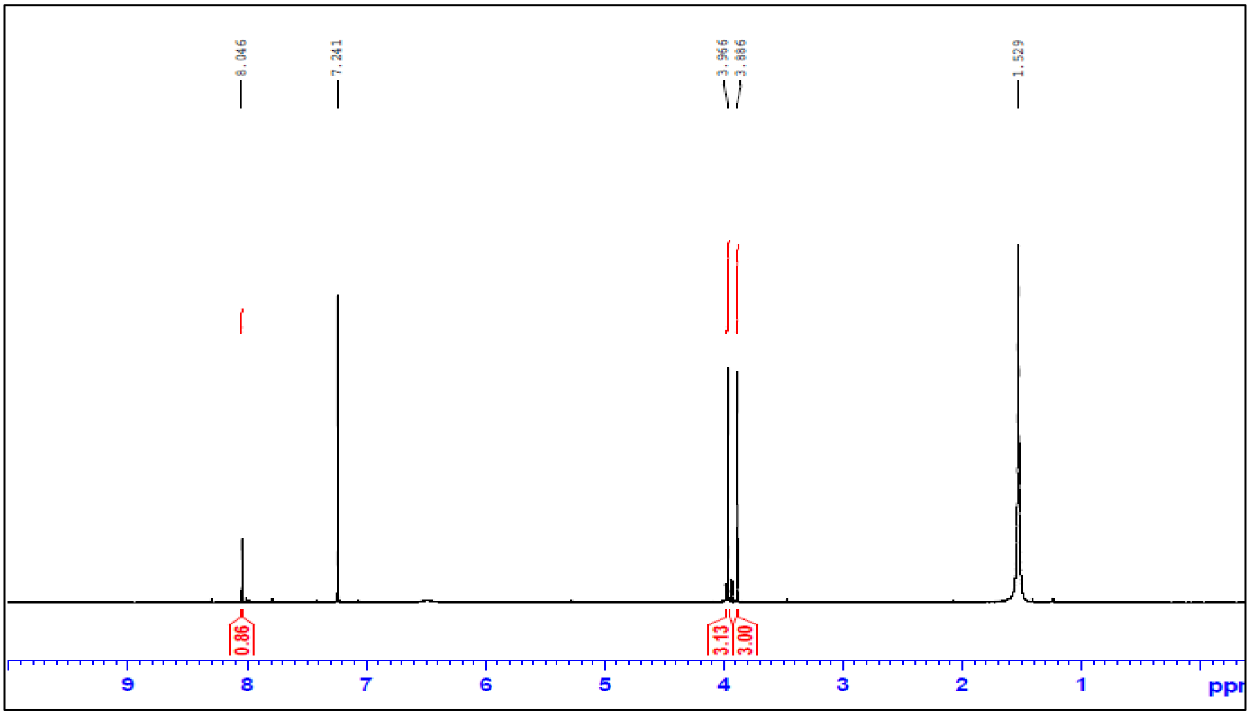

^{1}H NMR (600 MHz) of compound **4** in $CDCl_3$

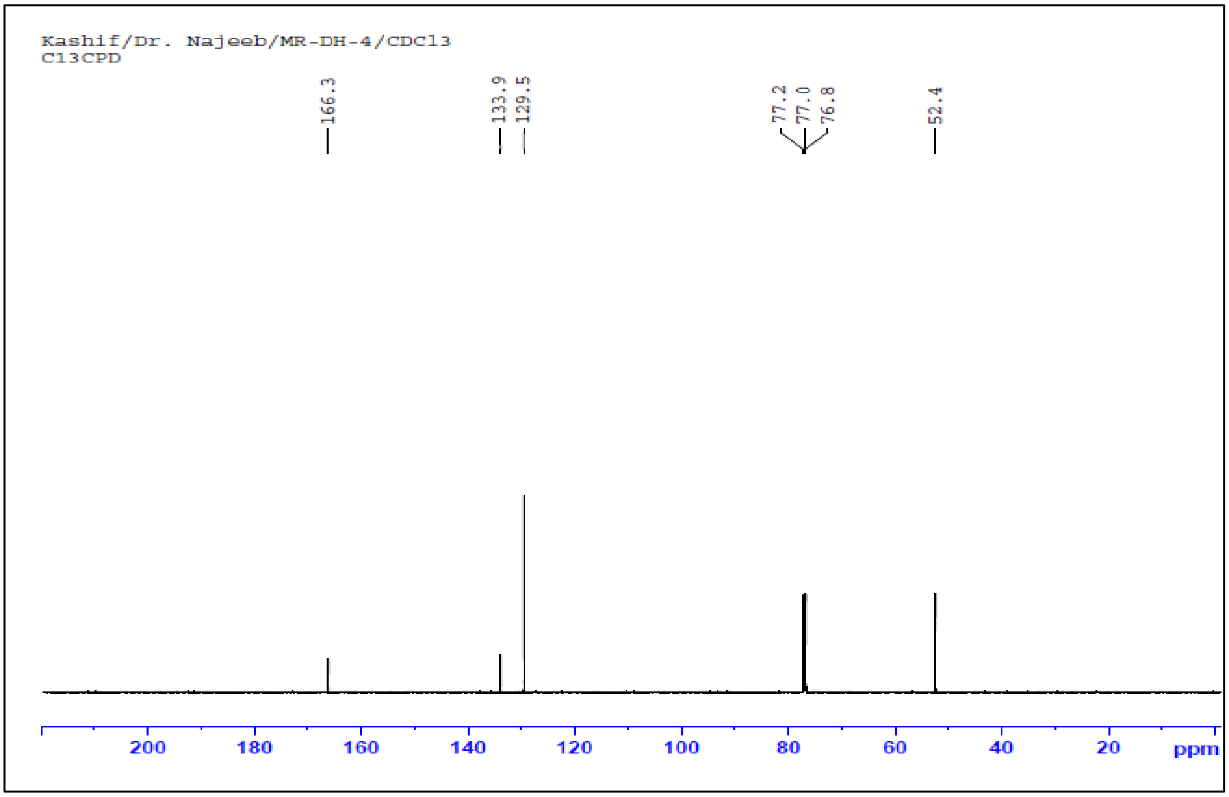

^{13}C NMR (125 MHz) of compound **4** in $CDCl_3$

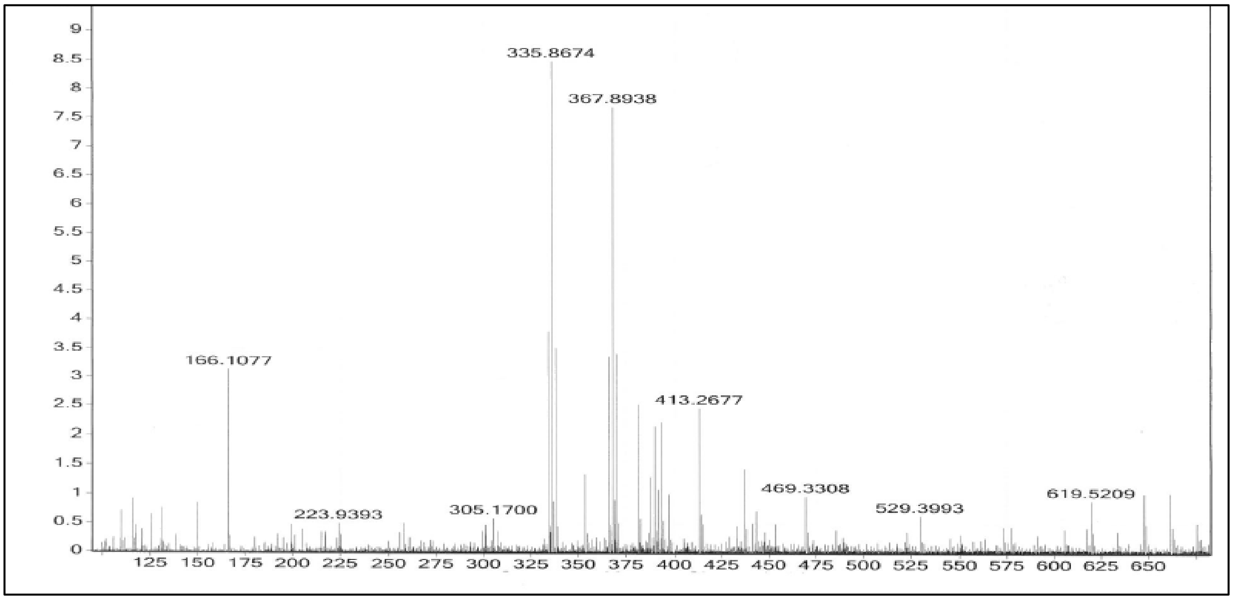

HR-ESI-MS (positive) of compound **4**

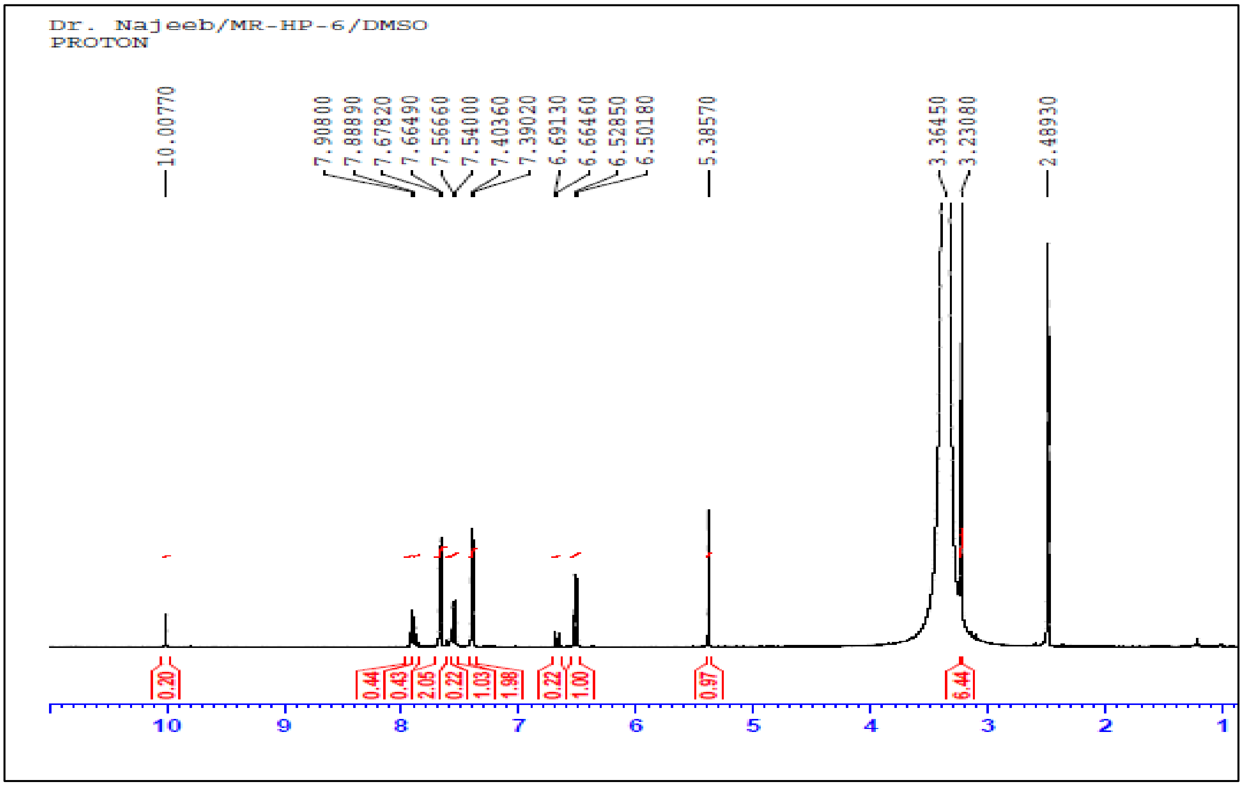

^{1}H NMR (600 MHz) of compound **5** in DMSO-*d*

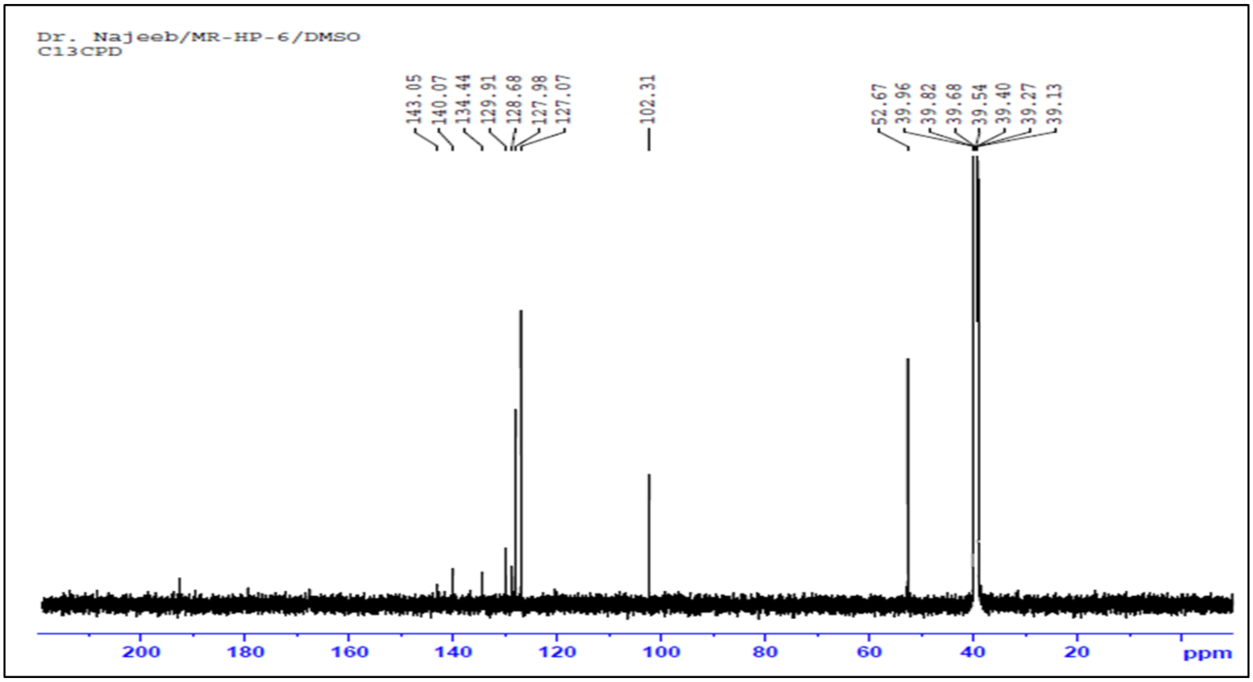

^{13}C NMR (125 MHz) of compound **5** in DMSO-*d*

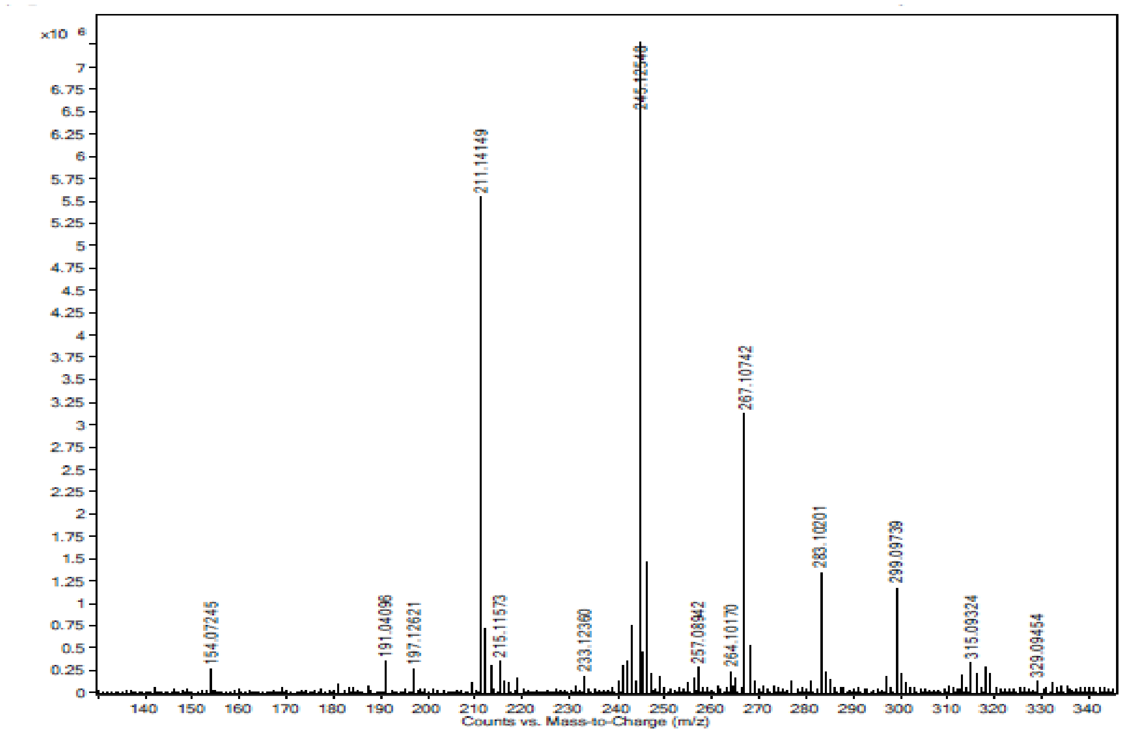

HR-ESI-MS (positive) of compound **5**

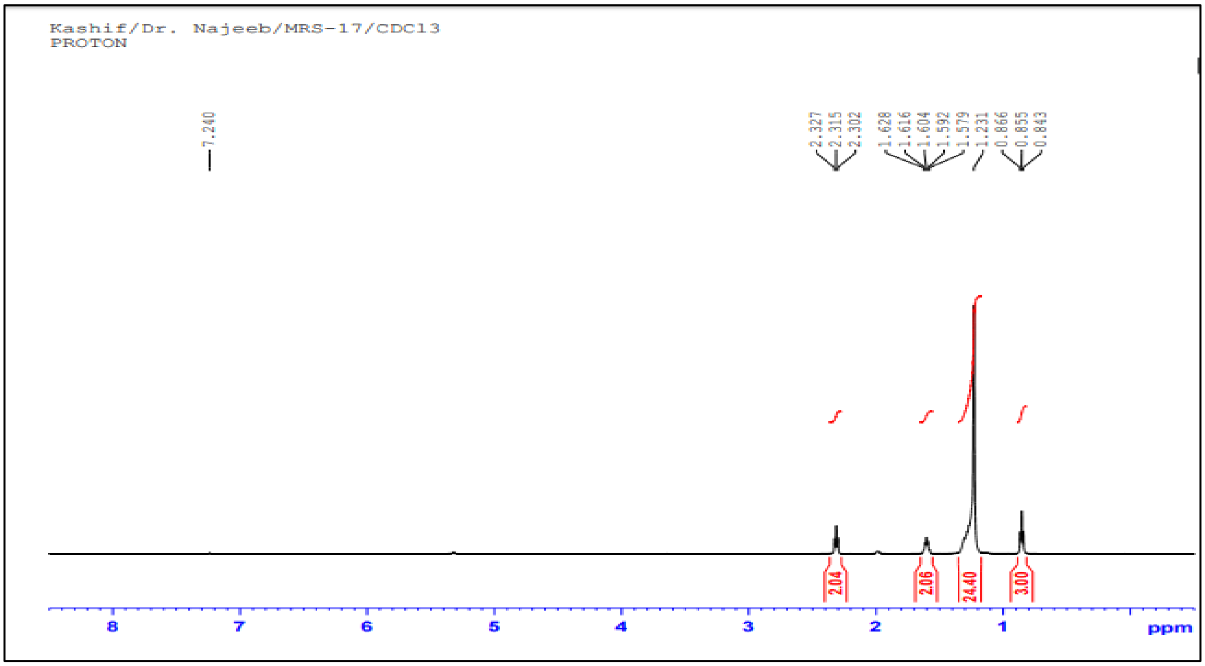

^{1}H NMR (600 MHz) of compound **6** in $CDCl_3$

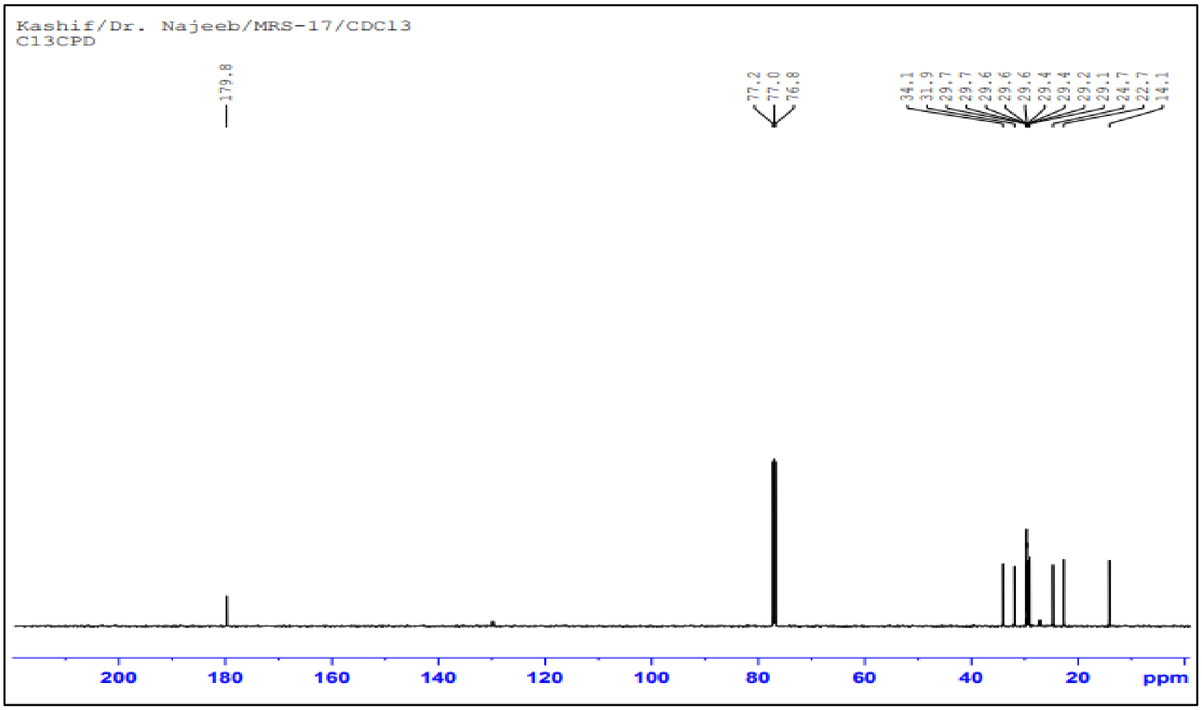

^{13}C NMR (125 MHz) of compound **6** in $CDCl_3$

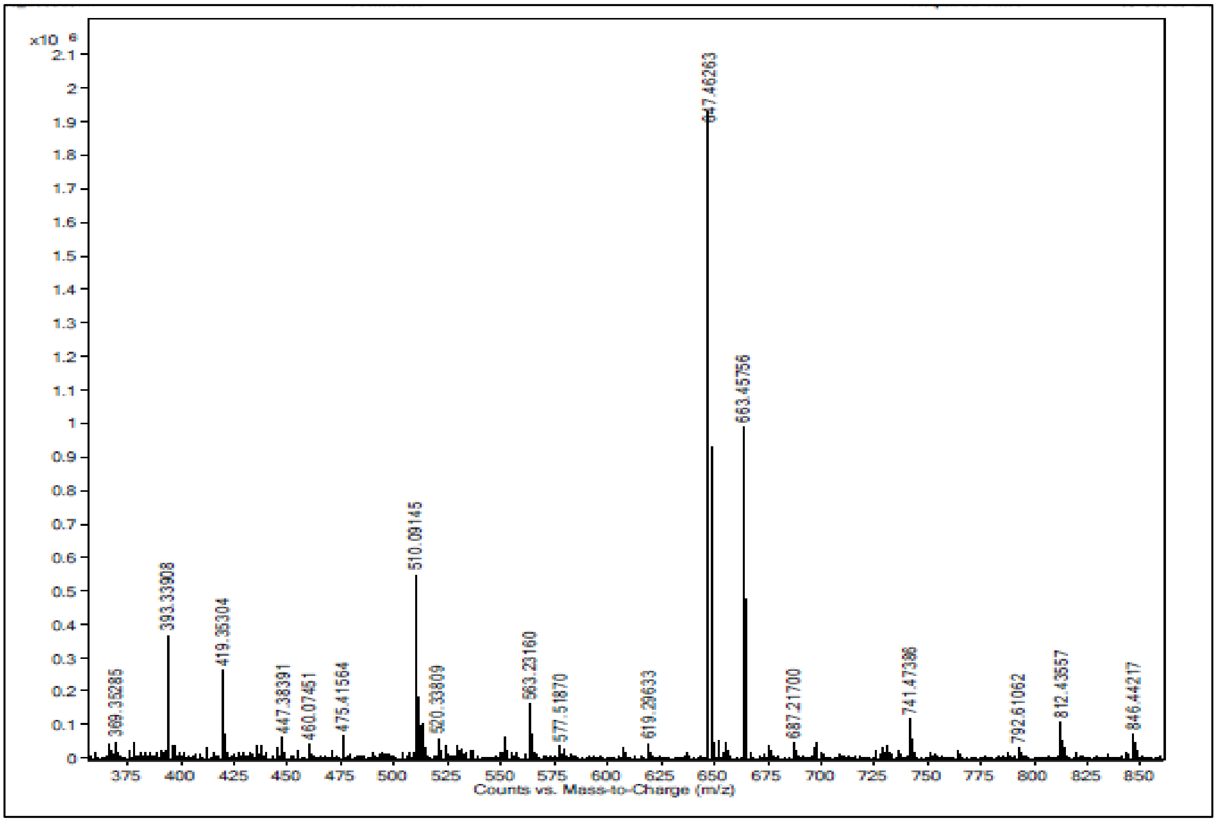

HR-ESI-MS (positive) of compound **6**

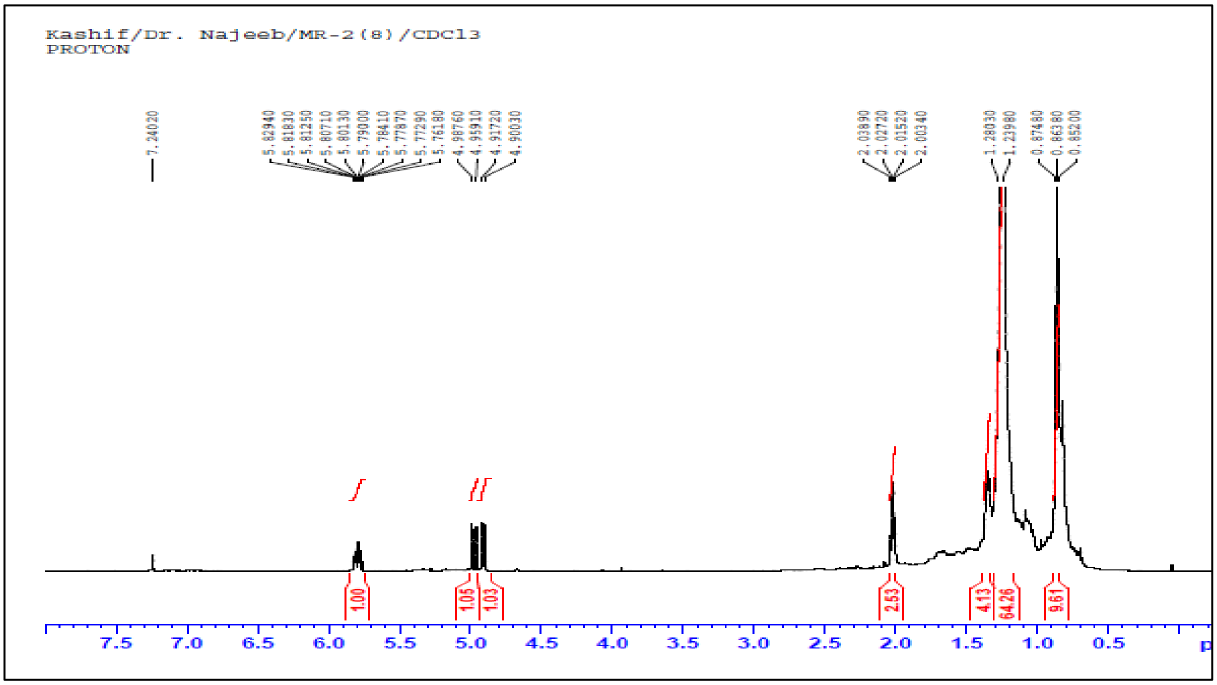

^1H NMR (600 MHz) of compound **7** in $CDCl_3$

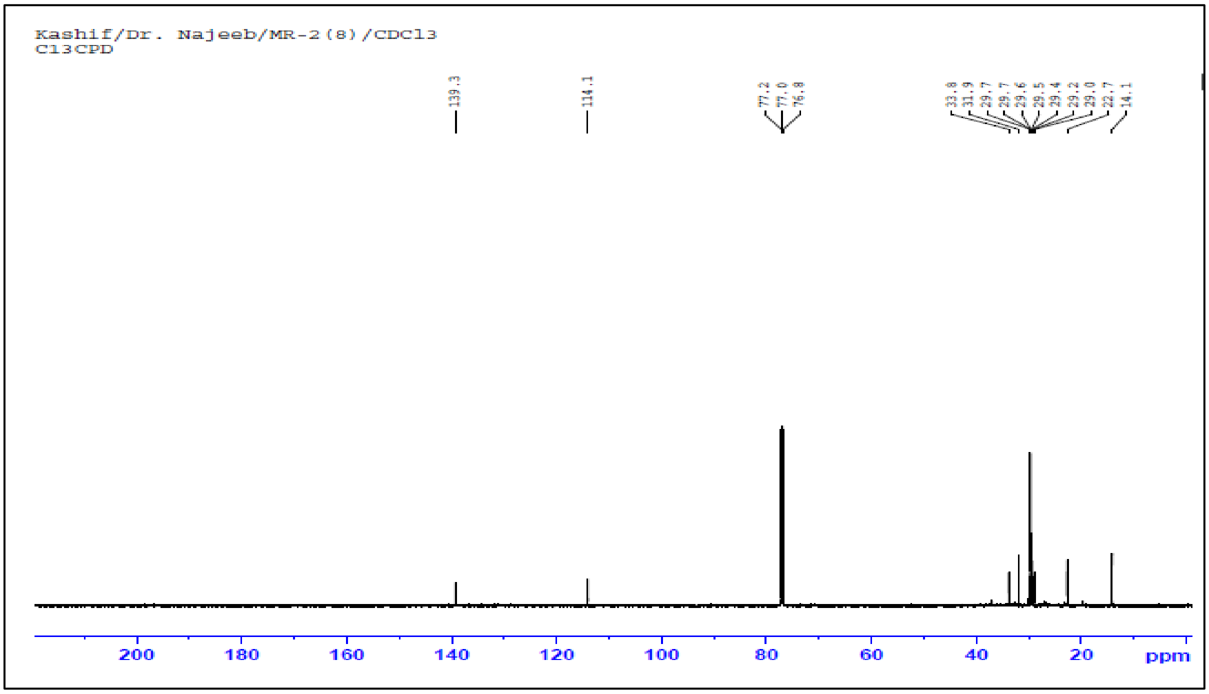

^{13}C NMR (125 MHz) of compound **7** in $CDCl_3$

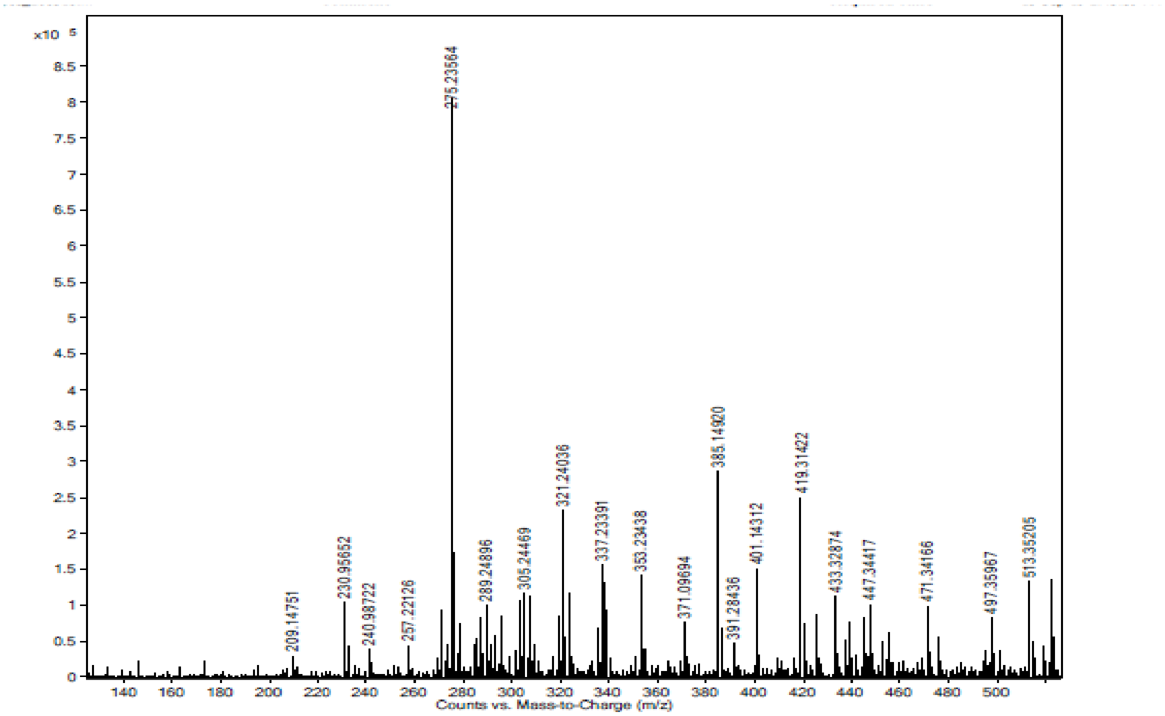

HR-ESI-MS (positive) of compound **7**

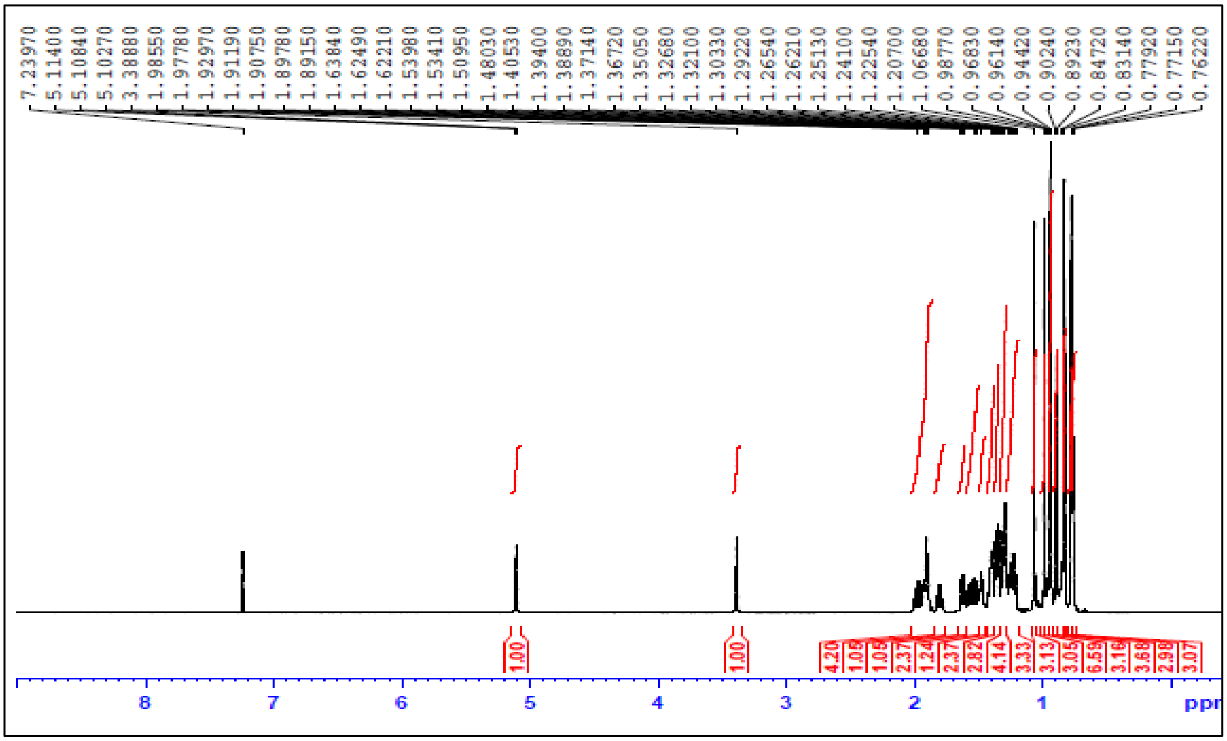

^{1}H NMR (600 MHz) of compound **8** in $CDCl_3$

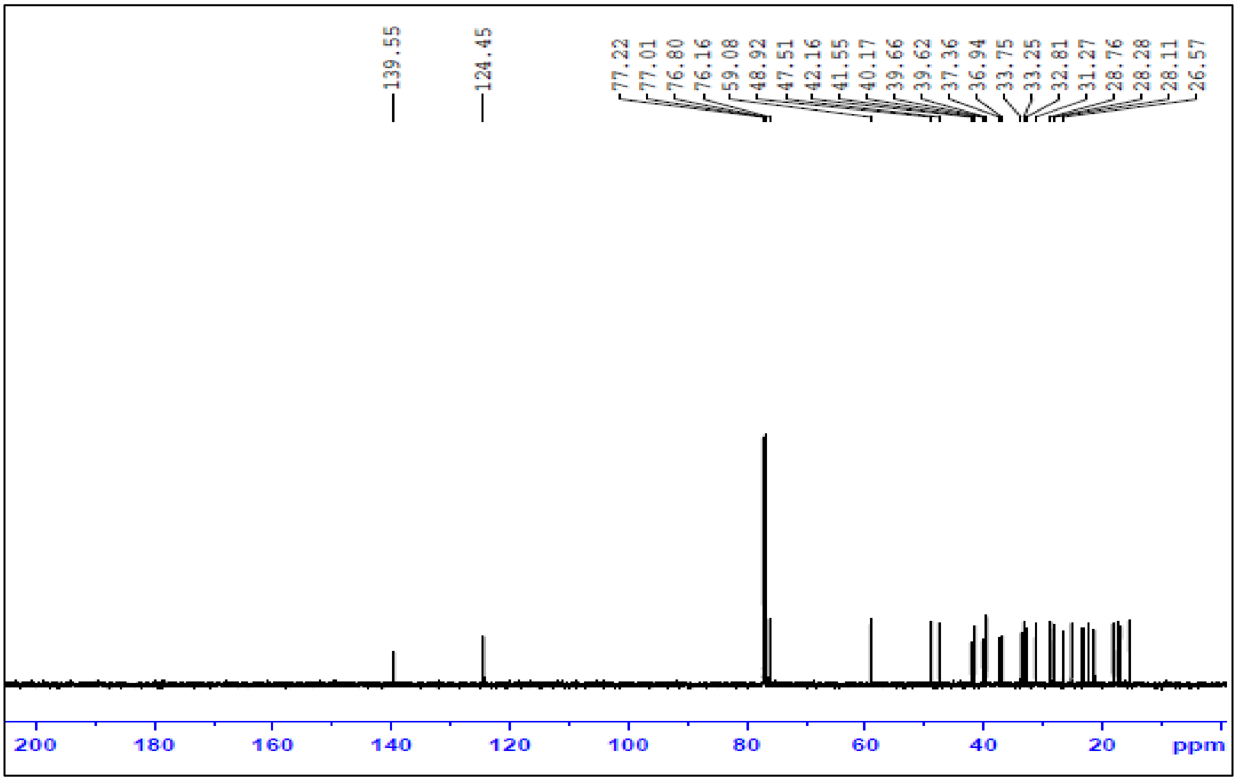

^{13}C NMR (125 MHz) of compound **8** in $CDCl_3$

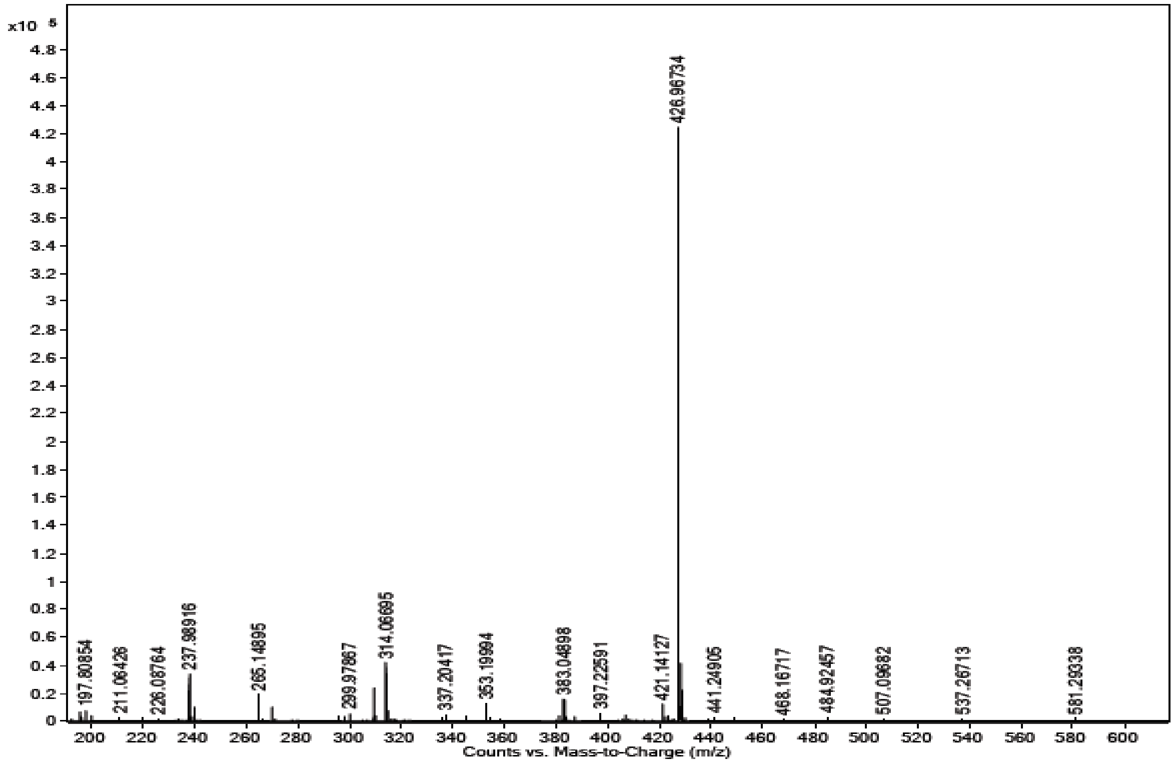

HR-ESI-MS (positive) of compound **8**

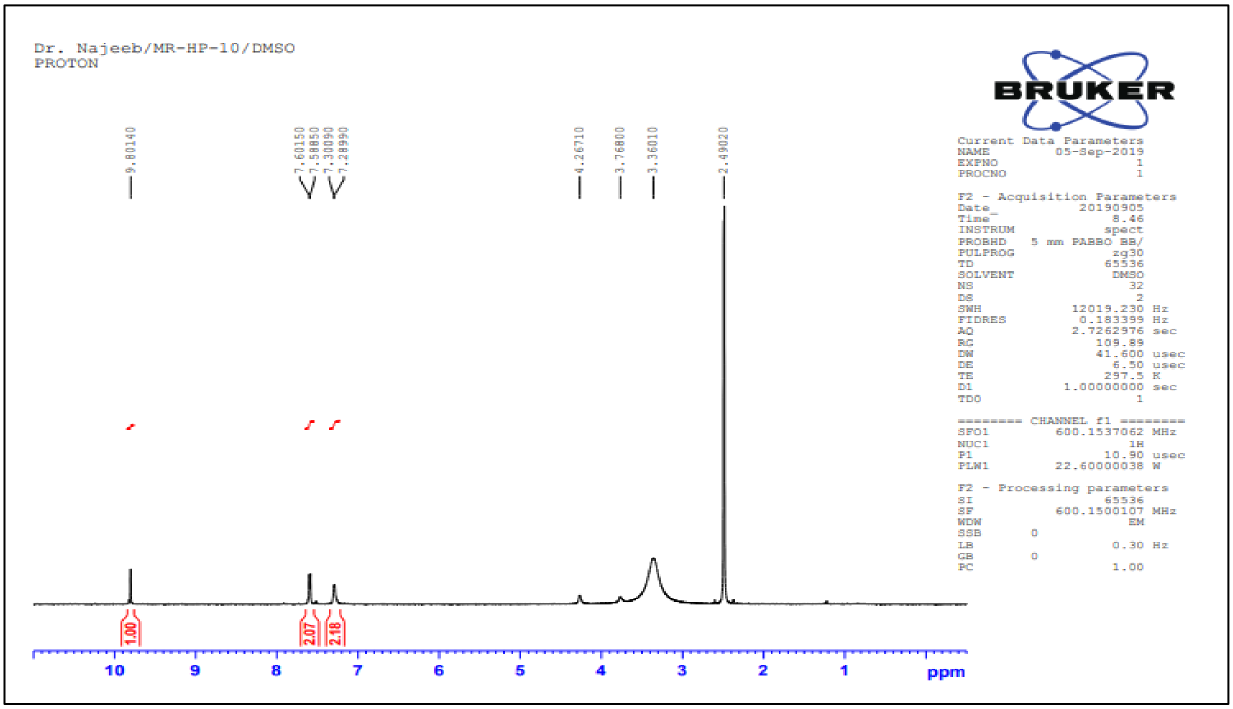

^{1}H NMR (600 MHz) of compound **9** in DMSO-*d*

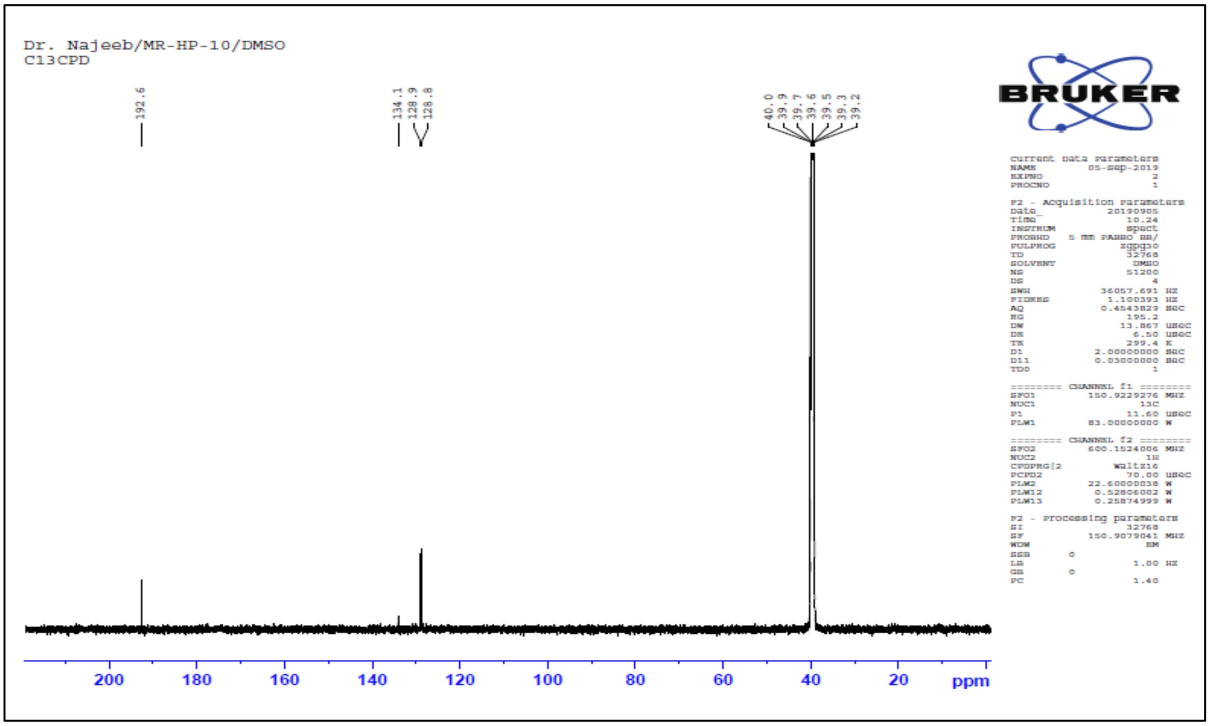

^{13}C NMR (125 MHz) of compound **9** in DMSO-*d*

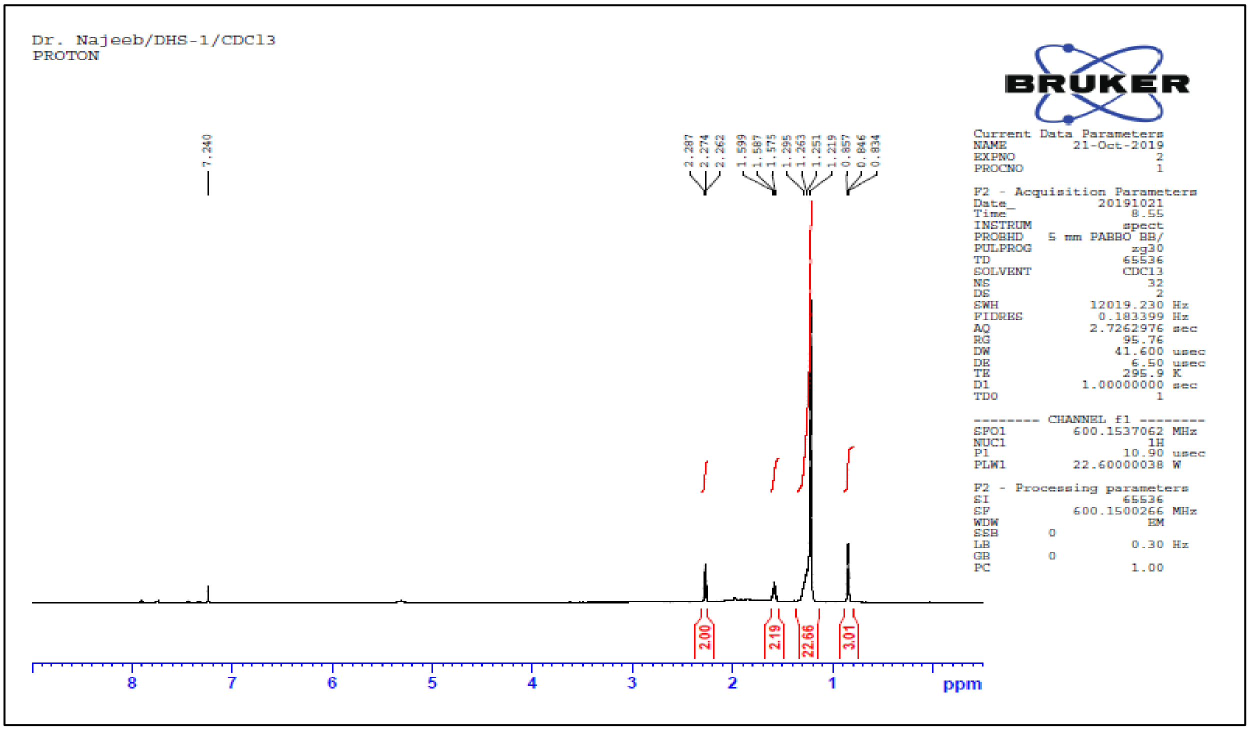

^{1}H NMR (600 MHz) of compound **10** in $CDCl_3$

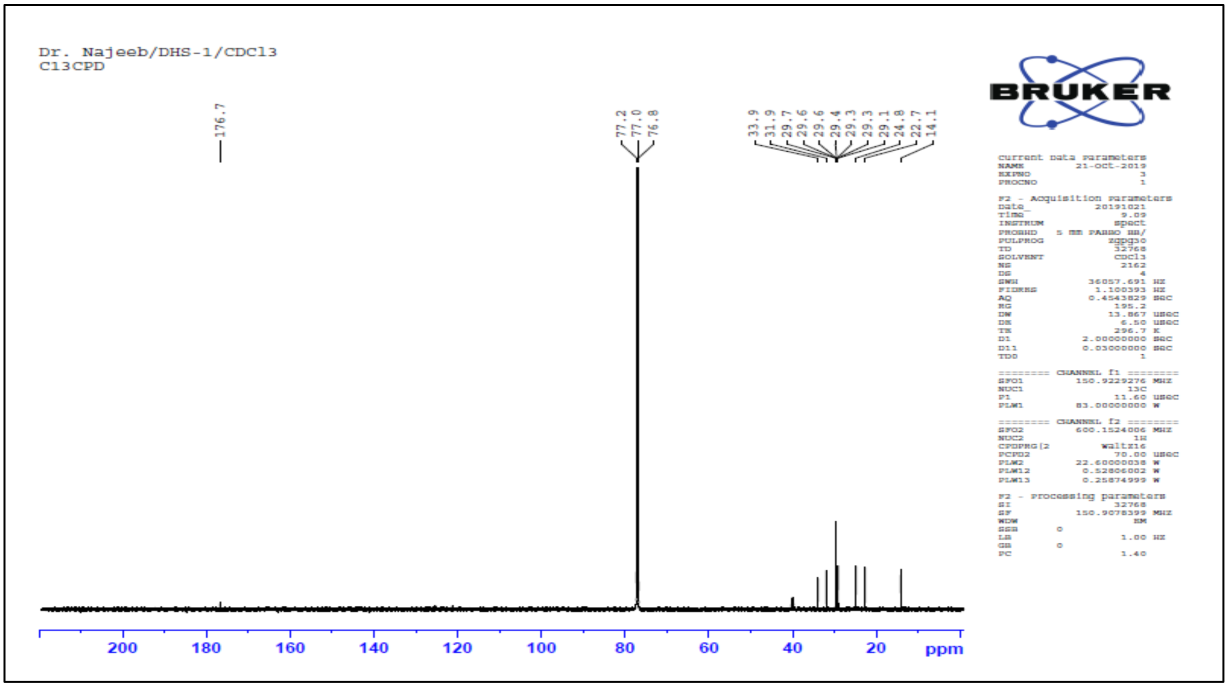

^{13}C NMR (125 MHz) of compound **10** in $CDCl_3$

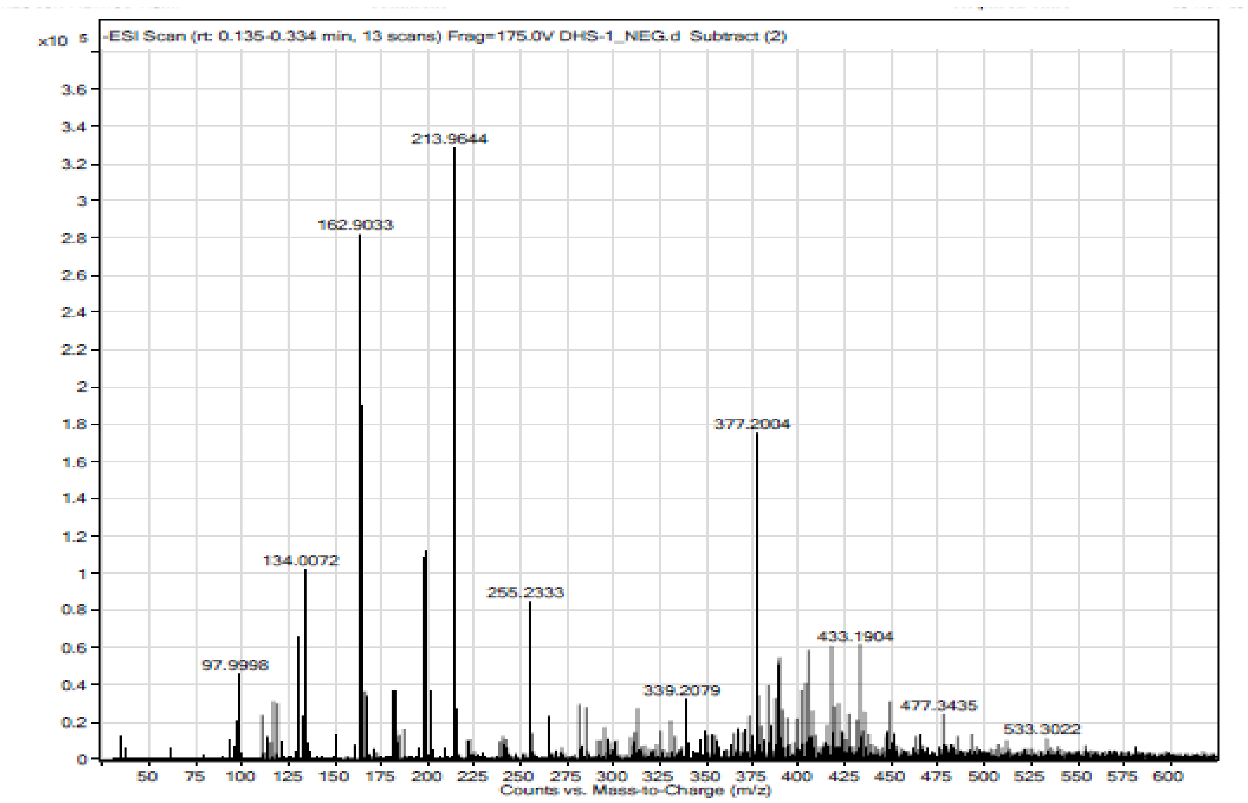

HR-ESI-MS (positive) of compound **10**

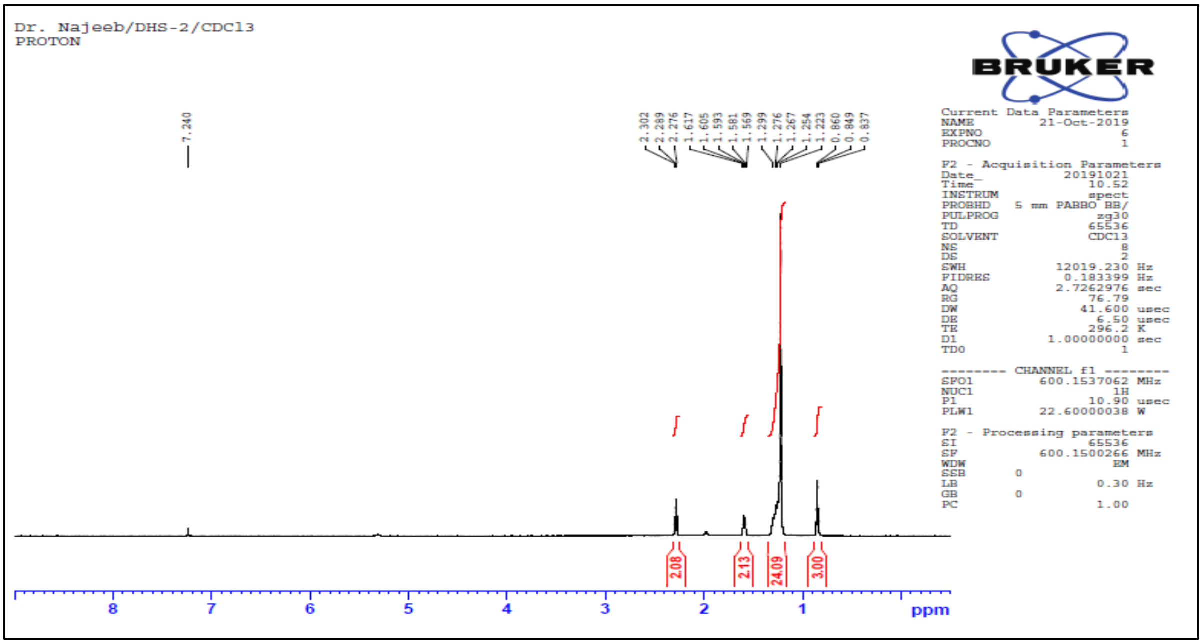

^{1}H NMR (600 MHz) of compound **11** in $CDCl_3$

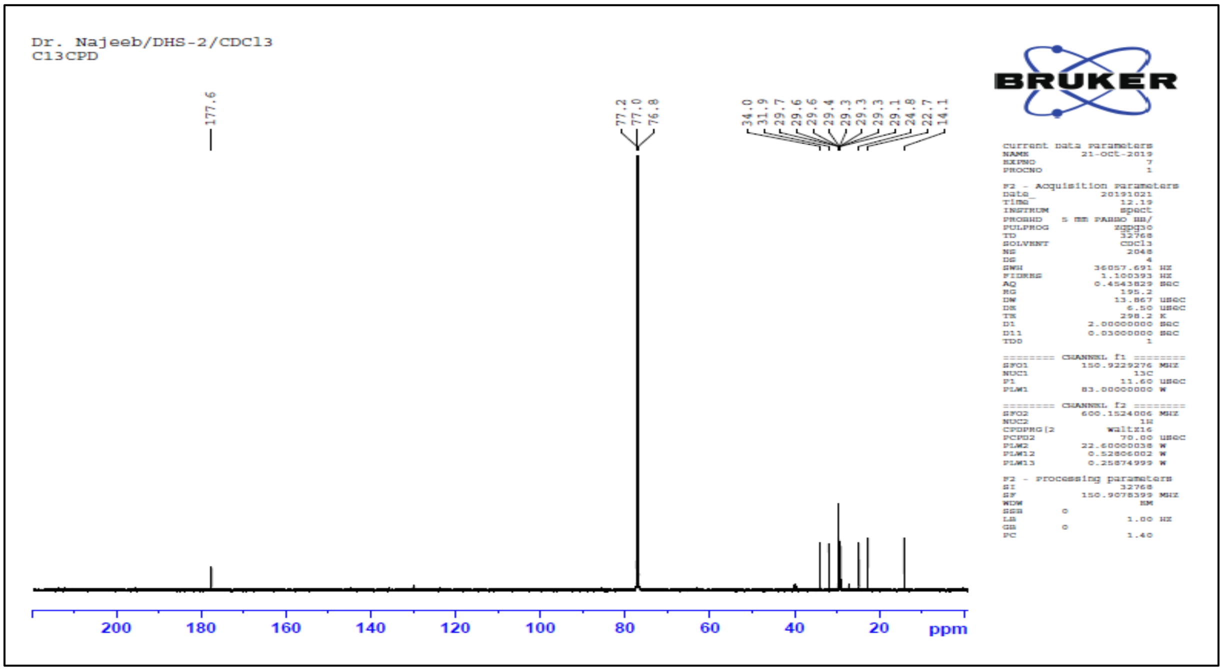

^{13}C NMR (125 MHz) of compound **11** in $CDCl_3$

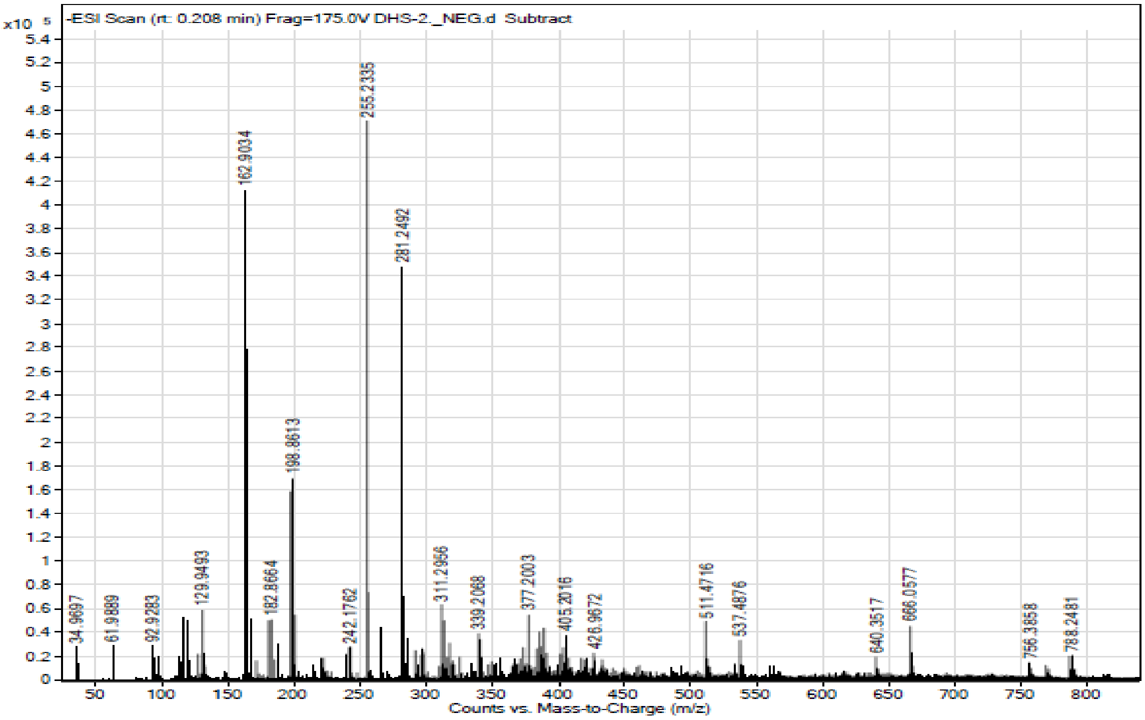

HR-ESI-MS (negative) of compound **11**

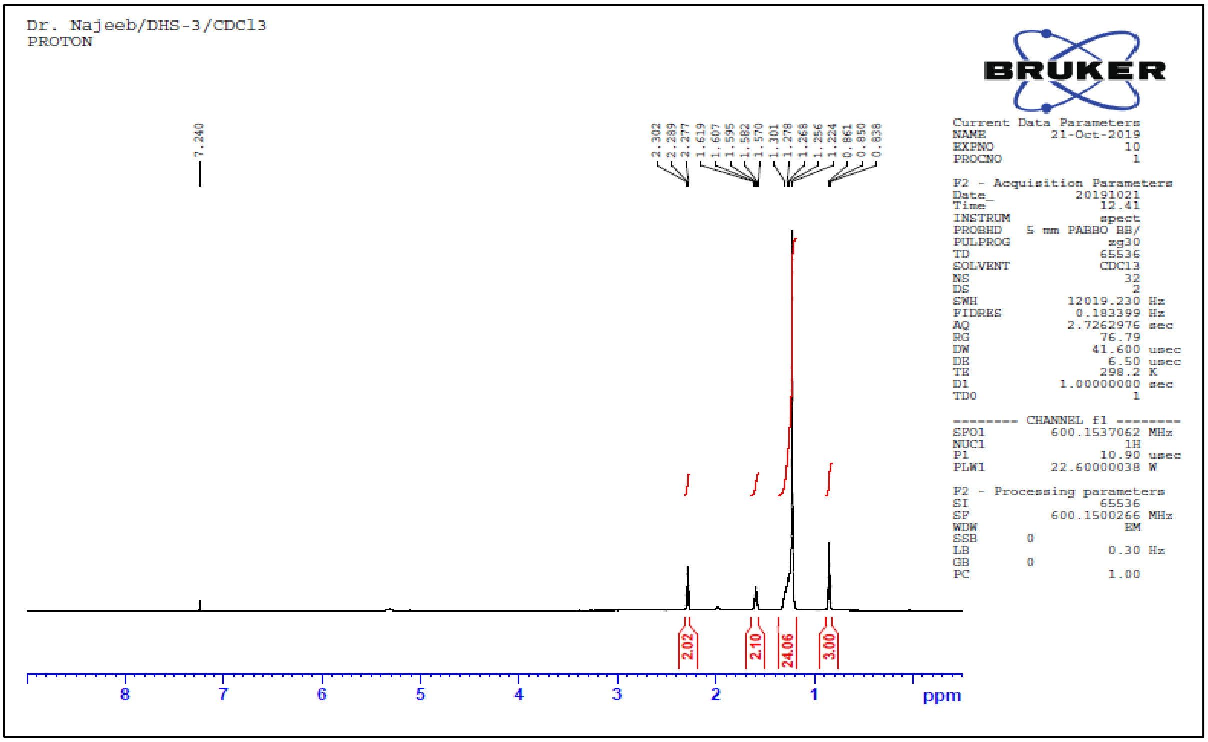

^{1}H NMR (600 MHz) of compound **12** in $CDCl_3$

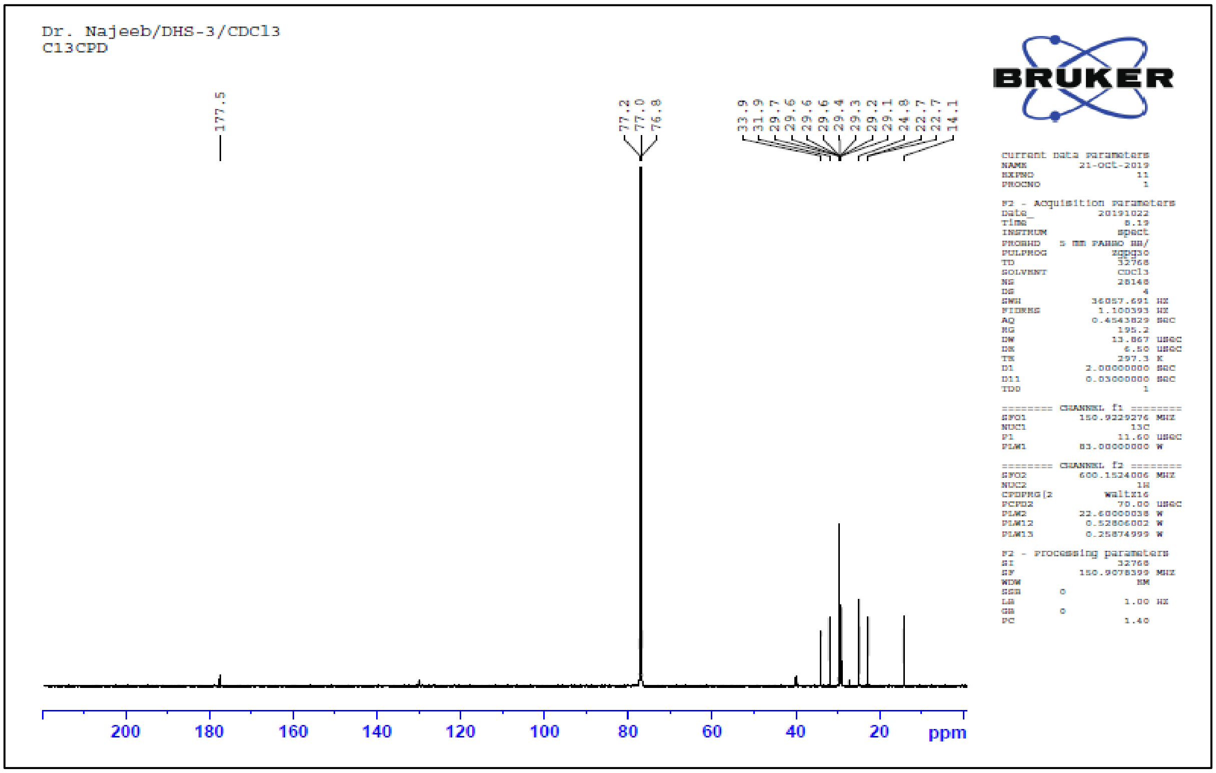

^{13}C NMR (125 MHz) of compound **12** in $CDCl_3$

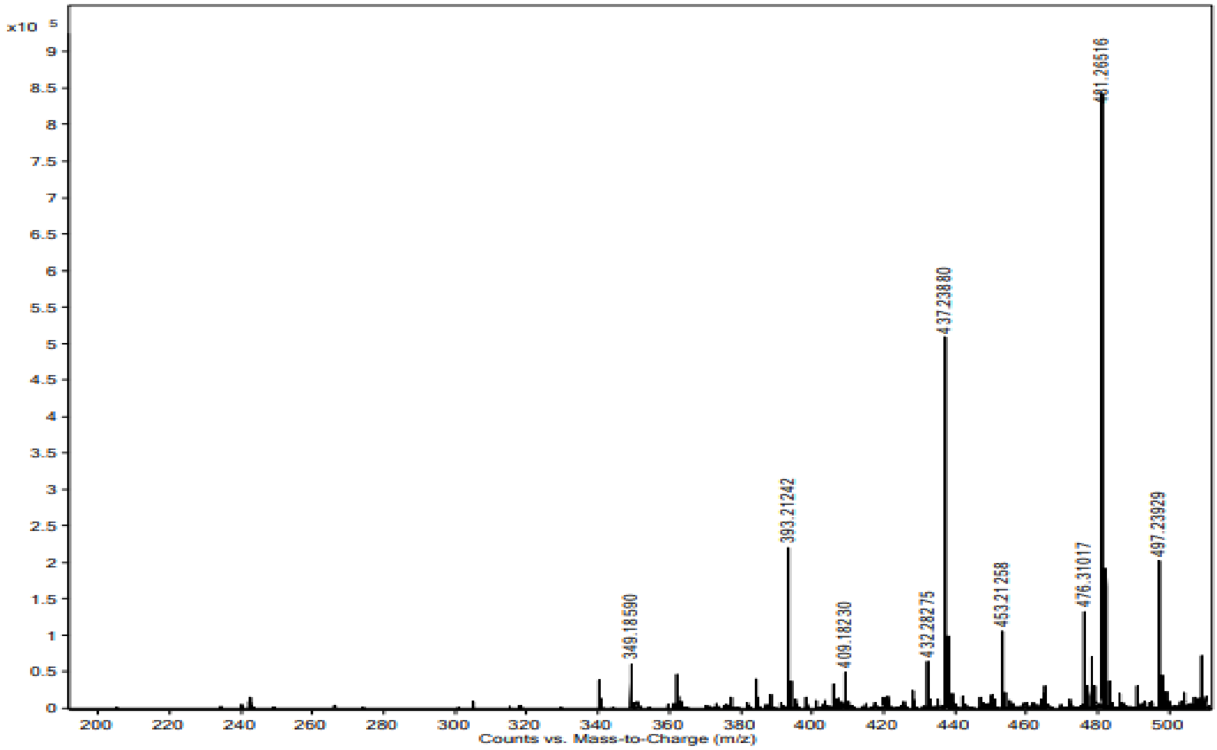

HR-ESI-MS (positive) of compound **12**

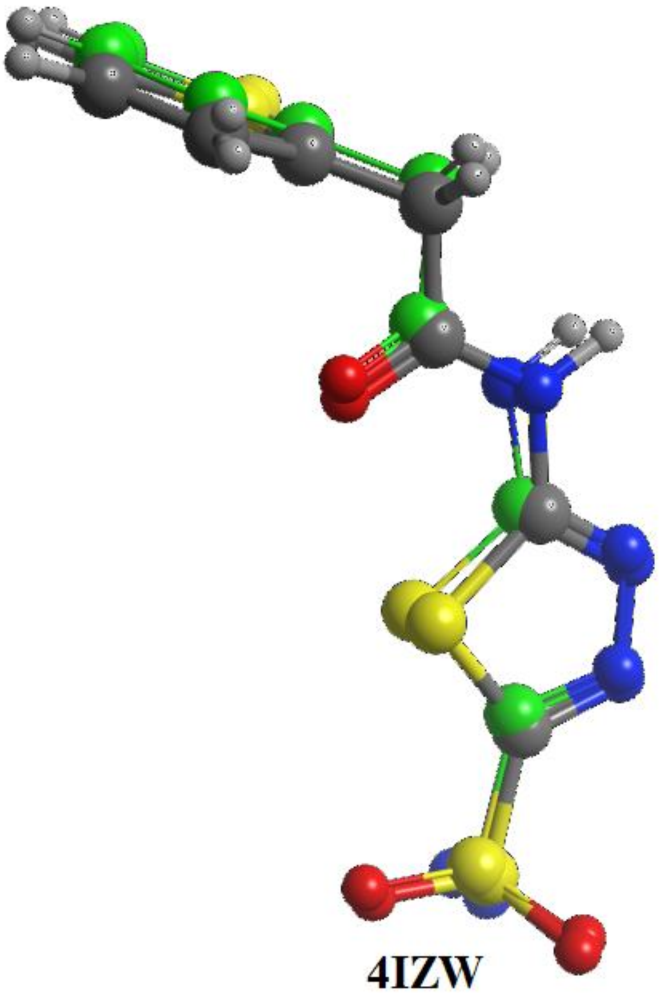

Figure S2. Superimposed view of co-crystalized ligand in 4IZW. The docked and the X-ray conformations of compound are shown in gray and green colors, respectively

Table S1. The predicted ADMET properties of compounds.

**Physicochemical Properties**													
**Comp**	**1**	**4**	**5**	**7**	**9**	**10**	**12**	**13**	**15**	**18**	**19**	**Reference**	**Acetazolamide**
Formula	C10H8O3	C11H11BrO4	C12H14O4	C17H34	C8H6O2	C22H44O2	C31H62O2	C11H11BrO4	C30H50O	C26H52O2	C28H54O2	C8H8N4O3S3	C4H6N4O3S2
MW	176.17 g/mol	287.11 g/mol	222.24 g/mol	238.45 g/mol	134.13 g/mol	340.58 g/mol	466.82 g/mol	287.11 g/mol	426.72 g/mol	396.69 g/mol	422.73 g/mol	304.37 g/mol	222.25 g/mol
NHA	13	16	16	17	10	24	33	16	31	28	30	18	13
NAHA	6	6	6	0	6	0	0	6	0	0	0	10	5
Fraction Csp3	0.00	0.27	0.25	0.88	0.00	0.95	0.97	0.27	0.87	0.96	0.89	0.12	0.25
NRB	3	4	5	14	2	20	29	5	5	24	25	5	3
HBA	3	4	4	0	2	2	2	4	1	2	2	6	6
HBD	1	0	1	0	0	1	1	0	1	1	1	2	2
MR	48.50	61.67	59.86	83.36	37.22	109.64	152.90	61.51	137.30	128.87	138.01	67.58	45.22
TPSA	54.37 Å^2	52.60 Å^2	55.76 Å^2	0.00 Å^2	34.14 Å^2	37.30 Å^2	37.30 Å^2	52.60 Å^2	20.23 Å^2	37.30 Å^2	37.30 Å^2	179.90 Å^2	151.66 Å^2
Lipophilicity													
Log $P_{o/w}$	1.27	2.76	2.09	4.78	1.03	5.26	7.15	2.85	5.11	6.23	6.45	0.26	-0.21
Water Solubility													
Log *S* (E SOL)	-1.89	-3.32	-2.12	-6.37	-1.57	-7.16	-10.43	-3.26	-7.98	-8.62	-9.04	-2.47	-1.14
Class	Very soluble	Soluble	Soluble	Poorly soluble	Very soluble	Poorly soluble	Insoluble	Soluble	Poorly soluble	Poorly soluble	Poorly soluble	Soluble	Very soluble
Pharmacokinetics													

GIA	High	High	High	Low	High	Low	Low	High	Low	Low	Low	Low	Low
BBBP	Yes	Yes	Yes	No	Yes	No	No	Yes	No	No	No	No	No
P-gpS	No	No	No	No	No	No	Yes	No	No	No	No	No	No
CYP1A2 Inh	No	Yes	No	Yes	Yes	Yes	No	Yes	No	No	No	No	No
CYP2C19 Inh	No	Yes	No	No	No	No	No	Yes	No	No	No	No	No
CYP2C9 Inh	No	No	No	No	No	No	No	No	No	No	No	No	No
CYP2D6 Inh	No	No	No	No	No	No	No	No	No	No	No	No	No
CYP3A4 Inh	No	No	No	No	No	No	No	No	No	No	No	No	No
Log K_p	-6.45 cm/s	-6.15 cm/s	-6.58 cm/s	-1.02 cm/s	-6.46 cm/s	-1.01 cm/s	1.68 cm/s	-6.14 cm/s	-2.34 cm/s	0.18 cm/s	0.39 cm/s	-7.41 cm/s	-7.84 cm/s
Druglikeness													
Lipinski	Yes	Yes	Yes	Yes; 1 violation: MLOGP >4.15	Yes	Yes; 1 violation: MLOGP >4.15	Yes; 1 violation: MLOGP >4.15	Yes	Yes; 1 violation: MLOGP >4.15	Yes; 1 violation: MLOGP >4.15	Yes; 1 violation: MLOGP >4.15	Yes	Yes
BS	0.85	0.55	0.85	0.55	0.55	0.85	0.85	0.55	0.55	0.85	0.85	0.55	0.55
Medicinal Chemistry													
PAINS	0 alert	0 alert	0 alert	0 alert	0 alert	0 alert	0 alert	0 alert	0 alert	0 alert	0 alert	0 alert	0 alert
Lead likeness	No; 1 violation: MW<250	Yes	No; 1 violation: MW<250	No; 3 violations: MW<250, Rotors>7,	No; 1 violation: MW<250	No; 2 violations: Rotors>7, XLOGP3>3.5	No; 3 violations: MW>350, Rotors>7,	Yes	No; 2 violations: MW>350, XLOGP3>3.5	No; 3 violations: MW>350, Rotors>7,	No; 3 violations: MW>350, Rotors>7,	Yes	No; 1 violation: MW<250

				XLOGP 3>3.5			XLOGP 3>3.5			XLOGP 3>3.5	XLOGP 3>3.5		
SA	2.03	2.13	2.31	2.71	1.00	3.00	4.11	2.02	6.27	3.49	4.26	3.00	3.00

BBBP = Blood Brain Barrier permeant, BS = Bioavailability Score , CYP = cytochrome P450, GIA = Gastrointestinal absorption HBA = Number of H-bond acceptors, HBD = Number of H-bond donors, Inh= Inhibitor, Log $P_{o/w}$ = Log P octanol/water, Log K_P = skin permeation, MR = Molar Refractivity, MW = Molecular weight, NAHA = Number of aromatic heavy atoms, NHA = Num. heavy atoms, NRB = Number of Rotatable bonds, P-gpS = P-glycoprotein substrate, Reference Ligand = (Co-crystallized Ligand), SA = Synthetic accessibility , TPSA = Topological Polar Surface Area
